# A prospective cohort study of SARS-CoV-2 infection-induced seroconversion and disease incidence in German healthcare workers before and during the rollout of COVID-19 vaccines

**DOI:** 10.1371/journal.pone.0294025

**Published:** 2024-01-30

**Authors:** Stephan Gehring, Frank Kowalzik, Omar Okasha, Tobias Engelmann, Daniel Schreiner, Christian Jensen, Aline Mähringer-Kunz, Wendy Hartig-Merkel, Thao Mai Phuong Tran, Cornelia Oostvogels, Thomas Verstraeten

**Affiliations:** 1 Zentrum für Kinder- und Jugendmedizin, Universitätsmedizin Mainz, Mainz, Germany; 2 P95 Pharmacovigilance and Epidemiology, Leuven, Belgium; 3 Klinik und Poliklinik für Radiologie, Universitätsmedizin Mainz, Mainz, Germany; 4 CureVac SE, Frankfurt, Germany; Universitas Syiah Kuala, INDONESIA

## Abstract

We assessed the seroepidemiology of SARS-CoV-2 infection and the incidence of coronavirus disease 2019 (COVID-19) before and during the rollout of COVID-19 vaccines, in a prospective observational cohort study on healthcare workers (HCWs) in a large tertiary hospital in Mainz, Germany. Antibody status was assessed during six visits between September 2020 and February 2022. Self-reported symptoms were collected using a smartphone application; symptomatic HCWs were tested using real-time polymerase chain reaction (RT-PCR) assays for SARS-CoV-2. Rates of virologically confirmed and severe COVID-19 were estimated using the U.S. Food and Drug Administration (FDA) and Coalition for Epidemic Preparedness Innovations (CEPI) case definitions, respectively, and were contrasted to background community transmission and circulating SARS-CoV-2 variants. A total of 3665 HCWs were enrolled (mean follow-up time: 18 months); 97 met the FDA definition of virologically confirmed COVID-19 (incidence rate (IR) 2.3/1000 person-months (PMs), one severe case). Most cases reported ≥2 symptoms, commonly, cough and anosmia or ageusia. Overall, 263 individuals seroconverted (IR 6.6/1000 PMs—2.9 times the estimated IR of COVID-19), indicating many cases were missed, either due to asymptomatic infections or to an atypical presentation of symptoms. A triphasic trend in anti-SARS-CoV-2 seroprevalence and seroconversion was observed, with an initial increase following the rollout of COVID-19 vaccines, a two-fold decline six months later, and finally a six-fold increase by the end of the study when Omicron was the dominant circulating variant. Despite the increase in infection rates at the end of the study due to the circulation of the Omicron variant, the infection and disease rates observed were lower than the published estimates in HCWs and rates in the general local population. Preferential vaccination of HCWs and the strict monitoring program for SARS-CoV-2 infection are the most likely reasons for the successful control of COVID-19 in this high-risk population.

## Introduction

In December 2019, the novel coronavirus SARS-CoV-2 was isolated from a cluster of patients presenting with pneumonia and who had epidemiological links to a seafood and wet animal market in Wuhan, Hubei Province, China [1]. The World Health Organization (WHO) designated the disease caused by SARS-CoV-2 as COVID-19, and on 12 March 2020, it was declared a pandemic [2]. As of 14 July 2022, more than 555 million cases were confirmed globally, and the number of deaths exceeded 6.3 million [3]. In Europe, more than 233.5 million confirmed cases and more than 2 million deaths were reported, of which 12.4% and 7.1% were reported in Germany, respectively [3].

Globally, multiple SARS-CoV-2 variants have been identified by genomic sequencing, and these have been classified as variants of concern (VOCs) according to their potential threat of increased transmission, severity, or immune escape. To date, five variants have been defined as VOCs—Alpha (B.1.1.7), Beta (B.1.351), Gamma (P.1), Delta (B.1.617.2), and Omicron (B.1.1.529) [4]. Additionally, seven subvariants of the Omicron VOC are currently listed by the WHO as lineages under monitoring (LUM), including subvariants BA.2 and BA.5. Early data showed that all VOCs carried a higher risk of hospitalization, intensive care unit (ICU) admission, and death than the wild-type virus, and that variants Beta and Delta carried a much higher risk of these outcomes than the other variants [5]. However, the risk of severe outcomes was later shown to be substantially lower for Omicron than for Delta, albeit with significant variation with age [6]. Recent data from the Robert Koch Institute (RKI) in Germany indicate that since 2021, three VOCs were dominant in Germany: Alpha (weeks 8 to 24, 2021), Delta (weeks 24 to 51, 2021), and Omicron (week 52,2021 to date) [7].

A variety of mitigation measures have been introduced to prevent the collapse of health systems, including efforts to control transmission, healthcare-focused measures, and economic support policies [8]. However, mitigation measures cannot be sustained for extended periods given their economic implications. Currently, the number of available drug treatments for COVID-19 is limited and the need for more effective ones remains. Meanwhile, evidence continuously emerges from randomized clinical trials on existing and new treatments, and recommendations are dynamically updated [9]. Therefore, even if vaccine effectiveness against different SARS-CoV-2 variants varies [10], vaccines are the cornerstone to overcoming this global pandemic, and the development of variant-updated vaccines is ongoing [11]. As mass vaccination programs are implemented worldwide, data analyzing their effects are urgently needed to provide a better understanding and assessment of such endeavors. As of 14 July 2022, there are 40 approved COVID-19 vaccines, 11 of which have been granted emergency use authorization [12]. In Germany, COVID-19 vaccination started at the end of December 2020 with the prioritization of high-risk groups, including HCWs. The European Medicines Agency and Paul Ehrlich Institute (PEI) approved vaccines during the study period were Comirnaty® (including vaccines with reduced dose and variant-specific vaccines), Jcovden® (COVID-19 Vaccine Janssen), Nuvaxovid® (NVX-CoV2373), Spikevax® (COVID-19 Vaccine Moderna, including vaccines with reduced dose and variant-specific vaccines) and Vaxzevria® (COVID-19 Vaccine Astra-Zeneca). More than 76% of the German population were fully vaccinated by 14 July 2022, and more than 61% received one booster dose or more [13].

Individual risk factors for a severe course of COVID-19 have been widely discussed. Diabetes mellitus, arterial hypertension, obesity, and cardiovascular or pulmonary disease are associated with higher morbidity and mortality rates in patients with SARS-CoV-2 infection [14–16]. Especially, disorders of the cardiovascular systems such as arterial hypertension were noted more frequently in severe COVID-19 patients compared to non-severe patients [17]. Similarly, a higher risk of disease progression to severe COVID-19 of patients with asthma and chronic obstructive pulmonary disease is discussed [18, 19]. Certain types of immunodeficiency were shown to increase the risk for severe COVID-19 [20]. Even associations with thyroid disease are described for the novel coronavirus [21]. Most of these are not only mere statistical associations but seem to be mediated by preexisting pro-inflammatory conditions [16, 21, 22]. Besides individual risk factors, the understanding of virus transmission and infection epidemiological data is pivotal in fighting a pandemic disease.

A large prospective cohort study in the UK and USA including persons who self-reported a positive COVID-19 test during the first wave (March to April 2020) estimated that HCWs had at least a three times higher risk of SARS-CoV-2 infection than the general population [23]. This occupational group is at increased risk of infection given their exposure to both hospitalized COVID-19 patients and community transmission [24]. Additionally, subclinical infections were shown to be significant, with up to 40% of SARS-CoV-2 infected HCWs being asymptomatic at the time of screening [25]. Although HCWs are considered a priority group for COVID-19 vaccines, vaccine hesitancy remains a significant challenge for ongoing immunization programs [26].

Given the rapidly changing situation and the urgent need to accelerate the licensing of efficacious candidate vaccines against SARS-CoV-2 infection, the present study aimed to assess virologically confirmed COVID-19 disease and infection-induced seroconversion in a cohort of HCWs at a tertiary care center in Germany before and during the rollout of COVID-19 vaccines. As SARS-CoV-2 variants keep evolving and novel viral infections may emerge in the future, our study provides urgently needed data on the effects of mass vaccine rollout in a pandemic setting. Healthcare workers who participated in our study were invited to be enrolled in a phase III COVID-19 vaccine trial (CureVac SE; ClinicalTrials.gov identifier: NCT04674189). The present study was aligned with the trial to provide additional controls for the exploratory efficacy analyses thereof.

## Materials and methods

### Study design, setting, and period

This was a prospective, observational, cohort study at the University Medical Centre of Mainz, Germany (UM Mainz), carried out between August 2020 and February 2022.

### Study population, inclusion, and exclusion criteria

A previous observational SARS-CoV-2/COVID-19 study conducted among the HCWs at UM Mainz, during spring 2020 (′CovidPreventMainz′ (CPM)) had enrolled a convenience sample of 3500 HCWs. Those individuals were the first to be offered enrollment in the present study and the initial targeted sample size of the present study was the same as in the ᾿CPM᾿ study i.e., 3500. After offering those individuals enrollment, other HCWs and medicine/dentistry students at UM Mainz were offered participation. These individuals, who were all above the age of 18 years, and who agreed to sign an informed consent form (ICF) were eligible for enrollment, which took place between August-October 2020. No exclusion criteria were defined. Approximately 35% of the present study population transferred into the COVID-19 vaccine trial from January 2021 onward.

### Definitions

A positive serostatus was defined as testing positive for anti-SARS-CoV-2 IgM and/or IgG antibodies, whereas a negative serostatus was defined as testing negative for both antibodies. Seroconversion was defined as a seronegative test at baseline and/or any of the follow-up visits, followed by a seropositive test. For the analyses of seroprevalence and seroconversion, a distinction was made between infection-specific serostatus, as defined by anti-nucleocapsid (anti-N) SARS-CoV-2 IgG antibodies, and vaccine-induced serostatus, as defined by anti-spike protein (anti-S) SARS-CoV-2 antibodies. However, the latter was still considered suggestive of infection-induced positive serostatus up to follow-up visit 3, given that COVID-19 vaccines were not authorized before that time. The definition of infection-induced positive serostatus at visits 4–6 was restricted to participants having only SARS-CoV-2 anti-N IgG antibodies, which are not elicited by vaccination.

Virologically confirmed COVID-19 disease was defined according to the U.S. FDA guidance as an acute illness with a positive SARS-CoV-2 RT-PCR test result and at least one symptom suggestive of COVID-19 (S1 Table). Secondary outcomes included the rates of COVID-19 disease based on the more specific CEPI case definitions for suspected COVID-19 disease and severe COVID-19 disease (S1 Table). The FDA, CEPI, and severe disease case definitions were chosen to be the same as those used in the anticipated vaccine trial (NCT04674189). A single episode of COVID-19 disease was defined by symptoms occurring between four days before the index RT-PCR test and four days after the last confirmatory RT-PCR test.

### Description of data collection

Study participants attended one baseline (visit 1) and five follow-up visits (visits 2–6), during which data on demographics, occupational information, risk factors, occurrence of any previous SARS-CoV-2 infection, and COVID-19 vaccination were collected (S2 Appendix). Participants were asked to indicate any disorder of the airways and/or lung, disorders of the cardiovascular system, or any immune deficiency. This did include a variety of diseases such as arterial hypertension, cardiovascular disease, asthma, or chronic obstructive pulmonary disease. Participants were not asked to provide further detail regarding their medical history or specify any reported disorders. After visit 1, the first three follow-up visits (visits 2–4) were scheduled every 6 weeks ±14 days, and the 5^th^ and 6^th^ visits were planned after 211 (±7 days) and 393 days (±21 days) after visit 3, respectively, to match the schedule in the vaccine trial (NCT04674189). Serum samples were collected at baseline and at all follow-up visits (S3 Table) for serological two-step testing: first, anti-N SARS-CoV-2 IgG antibodies were assayed using ARCHITECT® i2000SR by Abbott Laboratories (test sensitivity 100% and specificity 99.6%), and if positive or borderline-positive, samples were re-tested using Elecsys® Anti-N SARS-CoV-2 IgG/IgM by Roche Diagnostics (test sensitivity 99.5% and specificity 99.8%). The regimen for testing anti-S SARS-CoV-2 antibodies was adapted following the COVID-19 vaccine rollout to distinguish between vaccine-induced and infection-induced immunities, as shown in S4 Table.

In addition, self-reported data on symptoms suggestive of COVID-19 disease were collected via a mobile phone application (*TrialPal*®, Integra IT), first daily until alignment with the vaccine trial (i.e., up to visit 4) and twice weekly thereafter. The minimum compliance criterion for COVID-19 symptom reporting was once every two weeks. All study participants received automated push notifications as reminders, and those with multiple missing reports were contacted by the study site to ensure adherence. Participants who reported cough, shortness of breath, chills, smell or taste dysfunction, or fever (defined as a body temperature ≥ 37.8°C) were then screened by trained study personnel using scripted phone interviews to establish eligibility for virological confirmation. Eligible study participants were then invited to the study site for SARS-CoV-2 RT-PCR testing using self-collected nasopharyngeal swab samples. RT-PCR testing was performed using the Cobas-SARS-CoV-2 test (Roche), a single-well, dual-target PCR assay that includes both specific detection of SARS-CoV-2 and pan-Sarbecovirus detection for the Sarbecovirus subgenus family (which includes SARS-CoV-2). Testing was performed on the fully automated Cobas-6800/8800 Systems (Roche) according to the manufacturer’s instructions. Next, an algorithm was applied using the index test result (S1 Fig). Briefly, participants who tested positive for SARS-CoV-2 were interviewed to collect data, including data on the clinical course and outcome. Follow-up tests were scheduled after 21 days, then weekly until resolution, which was defined as two consecutive negative RT-PCR tests. Similarly, those who initially tested negative for RT-PCR were invited for a confirmatory test 24 hours after the index test.

### Protocol amendments

During the study period, the study protocol was amended three times and the ICF with the participants’ information sheet was amended twice, mostly to align the study with the vaccine trial. In brief, the first protocol amendment allowed for the inclusion of medicine and dentistry students, while the second and third amendments included an extension of the study period, the addition of two visits, and the specification of additional data collection about COVID-19 vaccination.

### Statistical analysis

We used descriptive statistics to summarize the demographic characteristics of the study population at visit 1. Seroprevalence at each visit was calculated as the percentage of participants who were seropositive divided by the total number at that visit. Seroconversion at each visit was calculated as the percentage of participants who had seroconverted at that visit divided by the total number of subjects at that visit who had been seronegative at the previous visit. The corresponding 95% confidence intervals (CIs) for seroprevalence and seroconversion were estimated using the Clopper-Pearson interval exact method [27]. To account for diagnostic misclassification, which can happen when diagnostic test characteristics (i.e., sensitivity and/or specificity) are less than 100%, we used the Bayesian approach proposed by Speybroeck et al. [28] to estimate the ‘true’ prevalence from the apparent ‘prevalence’. This approach was implemented in the *truePrev* function from the *prevalence* package in R. The IRs (per 1000 PM) of virologically confirmed COVID-19, including severe disease, were estimated as the number of confirmed cases divided by the total person-time at-risk. The person-time at-risk was calculated as the total follow-up time from baseline to end of study for participants without COVID-19 and from baseline to the date of virological confirmation of COVID-19 disease. The corresponding 95% CIs for the IR were computed using Garwood’s method [29]. A significance level of 5% was used for all analyses. All statistical analyses were carried out using R statistical software (R-4.0.4) [30].

### Ethical approval

The study was approved by the Independent Ethics Committee of the Medical Association of Rhineland-Palatinate (Landesärztekammer Rheinland-Pfalz), and all methods were in accordance with the Declaration of Helsinki. All study participants signed an ICF in German prior to participation. All signed and dated ICFs were available for verification by the study monitors.

### Study protocol registration

The study was registered in the European Union’s electronic register of Post-Authorization Studies (EU PAS register number EUPAS37174).

## Results

### Study enrollment and follow-up

A total of 3665 HCWs were enrolled in the study between August and October 2020 (S2A Fig). The study participants contributed about 40,000 PMs of follow-up, with a mean follow-up duration of approximately 12 months (median of 16.5 months, interquartile range (IQR) 4.8–17.4 months). The schedule of visits and number of subjects in each visit is shown in S2 Table. Participants who transferred to the vaccine trial, or discontinued for other reasons, had relatively shorter durations of follow-up. A total of 1227 subjects discontinued from the CPMprevac study (S6 Table). The main reason for study discontinuation was switching to the vaccine trial (66.6%), mostly between January and February 2021 (S2B Fig). The remaining reasons included ᾿lost to follow-up᾿ (n = 263, 21.4%), other reasons (n = 87, 7.1%), and withdrawal of participation (n = 57, 4.6%).

### Study population

The mean age of participants at enrollment was 39 years; more than 70% were younger than 50 years and more than 75% were females (Table 1). Nurses, doctors, and medical students represented 29.5%, 14.4%, and 16.6% of the study participants, respectively. Approximately 15% of participants were current or former smokers, ~17% had chronic lung and/or heart disease, ~14% reported they had work-associated direct contact with confirmed COVID-19 cases, and 10% reported contact with possible cases. There were no significant differences between participants who had a regular study end and those who discontinued, and the risk factor profile of the study population remained relatively unchanged throughout the study (Table 1).

**Table 1:** Baseline characteristics, including demographics and COVID-19 disease risk factors, among healthcare workers at Universitätsmedizin Mainz, Germany.

Characteristics	Category	All participants at enrollment (N = 3665) n (%)	Completed study (N = 2438) n (%)	Discontinued study (N = 1227) n (%)	p-value
Age group (years)	17–29	1110 (30.3)	756 (31.0)	354 (28.9)	0.99
	30–39	898 (24.5)	585 (24.0)	313 (25.5)	
	40–49	601 (16.4)	415 (17.0)	186 (15.2)	
	50–59	720 (19.6)	465 (19.1)	255 (20.8)	
	60–80	336 (9.2)	217 (8.9)	119 (9.7)	
Sex	Female	2761 (75.3)	1885 (77.3)	876 (71.4)	0.63
	Male	901 (24.6)	551 (22.6)	350 (28.5)	
	Other	3 (0.1)	2 (0.1)	1 (0.1)	
Occupational group	Doctor	529 (14.4)	358 (14.7)	171 (13.9)	0.27
	Nurse	1083 (29.6)	816 (33.5)	267 (21.7)	
	Student	608 (16.6)	383 (15.7)	225 (18.3)	
	Other	1445 (39.4)	881 (36.1)	564 (46.0)	
Smoker*	No	3110 (84.9)	2061 (84.5)	1049 (85.5)	0.99
	Yes	552 (15.1)	376 (15.4)	176 (14.3)	
Disorders of airways and/or lung **	No	3315 (90.5)	2193 (90.0)	1122 (91.4)	0.90
	Yes	344 (9.4)	241 (9.9)	103 (8.4)	
Disorders of cardiovascular system***	No	3362 (91.7)	2235 (91.7)	1127 (91.9)	1.00
	Yes	301 (8.2)	202 (8.3)	99 (8.1)	
Immune deficiency****	No	3568 (97.4)	2360 (96.8)	1208 (98.5)	0.76
	Yes	94 (2.6)	76 (3.1)	18 (1.5)	
Direct care for SARS-CoV-2 patients	No	3132 (85.5)	2027 (83.1)	1105 (90.1)	0.22
	Yes	528 (14.4)	407 (16.7)	121 (9.9)	
Aware of contact with a possible SARS-CoV-2 patient	No	3296 (89.9)	2176 (89.3)	1120 (91.3)	0.81
	Yes	369 (10.1)	262 (10.7)	107 (8.7)	
Possibly been in a region with known SARS-CoV-2 transmission++	No	94 (2.6)	76 (3.1)	18 (1.5)	0.76
	Yes	3568 (97.4)	2360 (96.8)	1208 (98.5)	
Swab/follow-up test for SARS-CoV-2 prior to the study+++	No	2501 (68.2)	1645 (67.5)	856 (69.8)	0.84
	Yes	1161 (31.7)	791 (32.4)	370 (30.2)	

*: 3, 1, and 2 participant(s) with missing information regarding smoking status, respectively for all participants, regular end participants, and discontinued participants.

**: 6, 4, and 2 participants with missing information regarding disorders of airways and/or lungs, respectively for all participants, regular end participants, and discontinued participants.

***: 2, 1, and 1 participant(s) with missing information regarding disorders of cardiovascular system, respectively for all participants, regular end participants, and discontinued participants.

****: 3, 2, and 1 participant(s) with missing information regarding immune deficiency, respectively for all participants, regular end participants, and discontinued participants.

+: 5, 4, and 1 participant(s) with missing information regarding direct care for COVID-19 patients, respectively for all participants, regular end participants, and discontinued participants.

++: 3, 2, and 1 participant(s) with missing information regarding possibly been in a region with known SARS-CoV-2 transmission, respectively for all participants, regular end participants, and discontinued participants.

+++: 3, 2, and 1 participant(s) with missing information regarding swab/follow-up test for SARS-CoV-2 prior to the study, respectively for all participants, regular end participants, and discontinued participants.

### COVID-19 vaccine uptake

**Fig 1:**
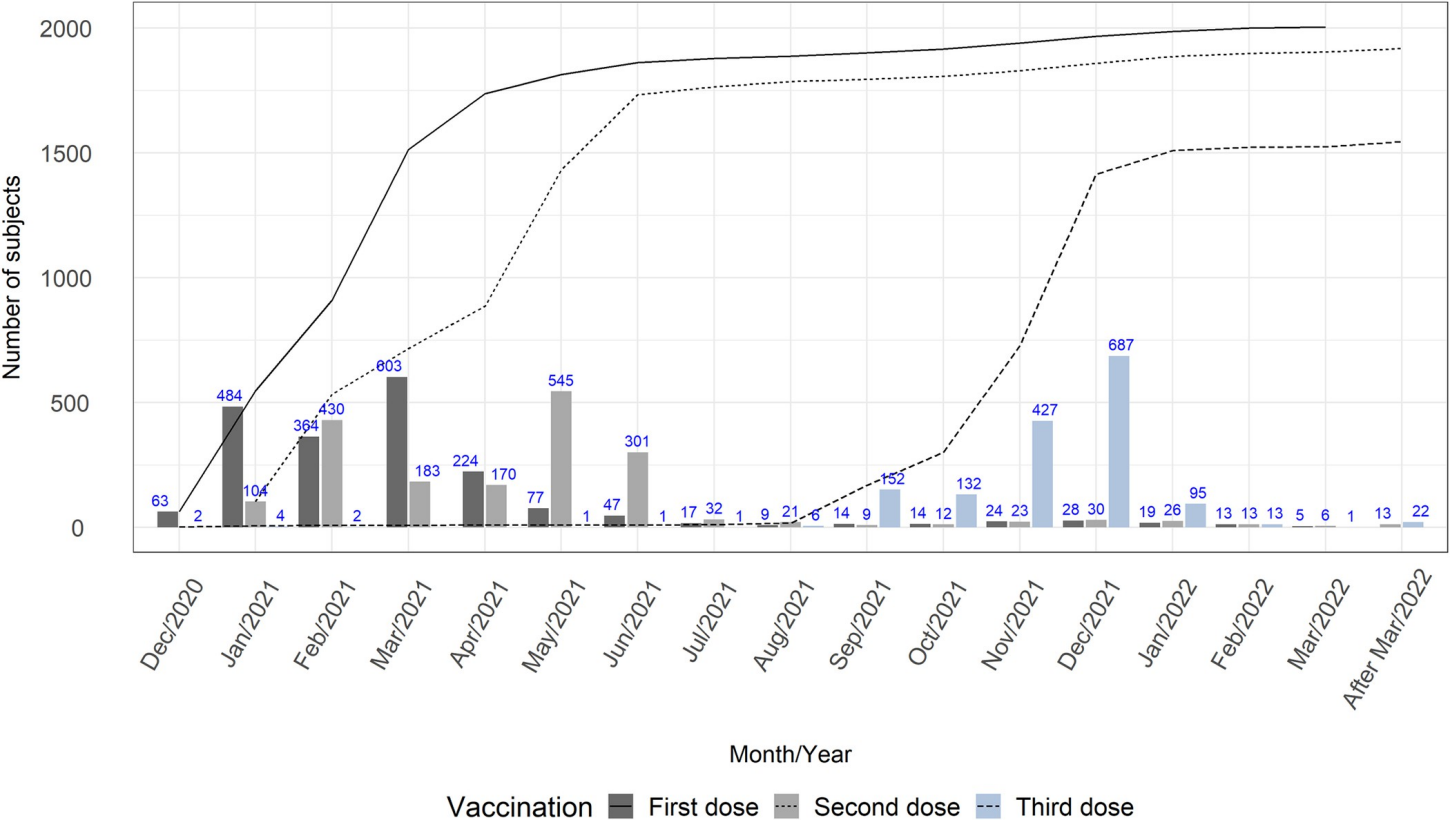
COVID-19 vaccine uptake among healthcare workers at Universitätsmedizin Mainz, Germany.

Between December 2020 and the study end (February 2022), 67.2% (2070/3082) of participants with complete follow-up and/or available vaccination data reported having received at least one dose of an authorized COVID-19 vaccine. Among 2438 subjects with complete follow-up, 1930 participants (79.2%) received at least one vaccination dose. Among these, 96.7% (1870/1930) and 81.8% (1578/1930) received at least two doses and three doses, respectively (Fig 1). The highest coverage of primary vaccination was reached by July 2021; COVID-19 vaccine boosting started in mid-2021, and its peak uptake was reached in January 2022. There were no significant differences in baseline characteristics between participants who were fully vaccinated, partially vaccinated, and unvaccinated, except for slightly older participants and slightly more males in the latter two groups (S7 Table).

### Seroepidemiology of SARS-CoV-2 infection

#### Seroprevalence

Of 3663 HCWs with available baseline serological data, 99 (2.7%; 95% CI: 2.2%-3.3%) were seropositive for anti-SARS-CoV-2 IgM and/or IgG antibodies (Table 2). Adjusting for diagnostic misclassification resulted in slightly lower seroprevalence estimates. Between visits 1 and 3, the seroprevalence rate increased from 2.7% to 4.3% and then doubled at visit 4, which coincided with the rollout of COVID-19 vaccines at UM Mainz. At visit 5, the seroprevalence rate reached more than 91%, consistent with the high vaccination coverage (Fig 1). However, the seroprevalence decreased to 14.4% at visit 6, the last follow-up visit. The seroprevalence at visit 3 was different between different age groups (S8 Table, panel A) with significantly higher seroprevalence among those aged less than 30 years old, compared to the other age groups. A similar pattern was observed at visit 4 in both vaccinated and unvaccinated participants (S8 Table, panel B). Moreover, the seroprevalence was significantly lower in the older age groups (50–59 and ≥ 60 years old), compared to the two younger age groups. At visit 5, the seroprevalence was not different between age groups, but was significantly higher in unvaccinated males compared to unvaccinated females (S8 Table, panel C).

**Table 2:** Crude and adjusted seroprevalence of anti-SARS-CoV-2 antibodies at scheduled visits 1–6.

	Visit 1 (baseline)	Visit 2	Visit 3	Visit 4	Visit 5	Visit 6
Total participants with serostatus data	3663	3615	3541	3275	2104	1880
Total participants with positive* serostatus	99	119	152	374	1919	271
Crude seroprevalence % [95% CI]	2.7 [2.2; 3.3]	3.3 [2.7; 3.9]	4.3 [3.6; 5.0]	11.4 [10.4; 12.6]	91.2 [89.9; 92.4]	14.4 [12.9; 16.1]
Adjusted** seroprevalence % [95% CI]	1.9 [1.8; 2.1]	2.5 [2.3; 2.7]	3.5 [3.3; 3.8]	10.7 [10.4; 11.1]	91.1 [90.7; 91.5]	13.8 [13.2; 14.3]

CI: confidence interval.

* Positive serostatus is defined as being seropositive for SARS-CoV-2 anti-N and/or anti-S antibodies. The latter does not differentiate infection-induced immunity from vaccine-induced immunity in the context of COVID-19 mRNA vaccines.

** Adjusted for the sensitivity and specificity of the test.

To separate infection-induced antibody response from vaccine-induced antibody response, the seroprevalence for anti-N and anti-S SARS-CoV-2 antibodies in visits 4–6 are presented separately in Table 3. The seroprevalence of anti-N antibodies first decreased from 1.3% to 0.4% between visits 4 and 5 i.e., a more than 3-fold decrease, and then increased from 0.4% to 6.2% between visits 5 and 6 (more than 15-fold). This increase in SARS-CoV-2 infection rates toward the end of the study corresponds with the circulation of the highly transmissible VOC Omicron. The seroprevalence of anti-S antibodies increased from 8.2% to 88.6%, i.e., more than 10-fold, between visits 4 and 5, which is consistent with the high COVID-19 vaccine uptake in the study population, and then decreased from 88.6% to 6.8% i.e., 13-fold, between visits 5 and 6.

**Table 3:** Anti-SARS-CoV-2 anti-N IgG and anti-S seroprevalence at scheduled visits 4, 5 and 6.

	Anti-N only*		Anti-S only**	
	Total number of participants with positive anti-N IgG but with negative anti-S IgG or IgM	Crude seroprevalence % [95% CI]	Total number of participants with positive anti-S IgG or anti-S IgM but with negative anti-N IgG	Crude seroprevalence % [95% CI]
Visit 4 N = 3275	42	1.3 [0.9; 1.7]	268	8.2 [7.3; 9.2]
Visit 5 N = 2104	9	0.4 [0.2; 0.8]	1864	88.6 [87.2; 89.9]
Visit 6 N = 1880	117	6.2 [5.2; 7.4]	127	6.8 [5.7; 8.0]

CI: confidence interval.

* Positive serostatus for anti-N IgG only.

** Positive serostatus for anti-S IgG/IgM only.

#### Seroconversion

The overall seroconversion rate during the study period was estimated to be 6.6 per 1000 PMs (95% CI: 5.8–7.5) (Table 4). Between visits 1–3, the seroconversion rate increased from 7.9 to 12.3 per 1000 PMs (i.e., a 1.6-fold increase). The rate then decreased by a factor of 7.2 between visits 3 and 5, the period during which COVID-19 vaccination was rolled out and reached peak uptake. However, the seroconversion rate again increased five-fold between visits 5 and 6 (from 1.7 to 8.5 per 1000 PMs). Except for age, participants who seroconverted for anti-N IgG antibodies were similar in terms of baseline characteristics to those who did not seroconvert throughout follow-up (S5 Table).

**Table 4:** SARS-CoV-2 seroconversion rates at scheduled visits 2 to 6.

	Anti-N IgG and/or anti-S IgM		Anti-N IgG**			Anti-S IgM at visits 2–3, and/or anti-N IgG at visits 2–6
	Visit 2	Visit 3	Visit 4	Visit 5	Visit 6	Overall
Population at risk*	3564	3496	3448	3169	2049	NA
Total participants who seroconverted	36	53	32	19	123	263***
Median follow-up time (months)	1.2	1.2	1.4	4.8	7.9	1.4
Total follow-up (person-months)	4,512	4,299	4,963	11,485	14,522	39,781
Seroconversion rate (per 1000 person-months) [95% CI]	8.0 [5.6; 11.1]	12.3 [9.2; 16.1]	6. 5 [4.4; 9.1]	1.7 [1.0; 2.6]	8.5 [7.0; 10.1]	6.6 [5.8; 7.5]

CI: confidence interval.

* Total number of subjects with negative serostatus at previous visit.

** At visits 4–6, seroconversion was restricted to positive anti-N IgG antibodies, which are specific for infection-induced immune response and, therefore, not subject to vaccine-induced immune response. Since COVID-19 vaccines were not yet available during visits 2–3, seroconversion was also defined as having positive anti-S IgM antibodies.

*** 255 participants seroconverted for anti-N IgG (at visits 2–6) and/or anti-S IgM (at visits 2–3), of whom eight seroconverted twice. Hence, there were 263 seroconversion events in total.

### COVID-19 disease

#### Incidence

**Fig 2:**
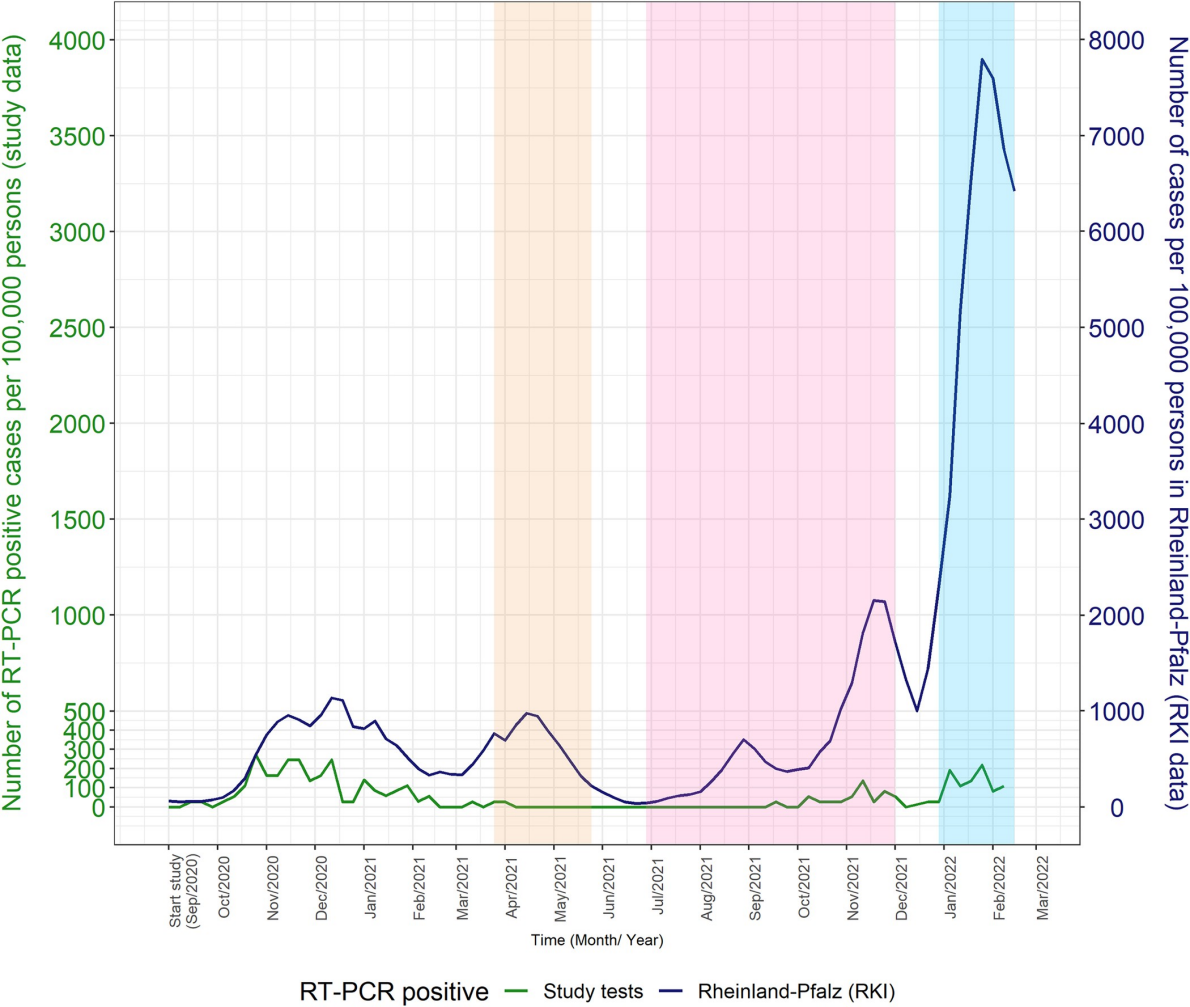
Comparison of SARS-CoV-2 incidence in this study and in the Rheinland-Pfalz region, Germany, based on RT-PCR positive results, showing internal tests only (solid green line) and both internal and external tests (blue dashed line). Rates in the Rheinland-Pfalz region are shown as a solid red line. The yellow shaded area indicates the period when the Alpha variant was dominant in Germany (i.e., comprising ≥80% of circulating variants), whereas the pink and blue shaded areas represent the periods when the Delta and Omicron variants were dominant, respectively. (RKI: Robert Koch Institute).

A total of 104 study participants tested positive for SARS-CoV-2 at UM Mainz, of whom 97 met the FDA definition of virologically confirmed symptomatic COVID-19 case (Table 5). The cumulative incidence and incidence density rates, based on the FDA definition, were 26.5 per 1000 participants and 2.3 per 1000 PMs, respectively. Slightly lower estimates were obtained using the more specific CEPI case definition (Table 5). Broadly, RT-PCR positive samples were clustered around three main peaks: the first peak coincided with follow-up visits 2–3 (i.e., pre-COVID-19 vaccination, around October and December 2020), the second peak was between visits 5 and 6; and the third peak was around the last visit around January-February 2022, i.e., about one year after COVID-19 vaccination (Fig 2). Of the 104 participants who had a positive RT-PCR result, one met the FDA definition of virologically confirmed severe COVID-19 and none met the CEPI case definition for severe disease (Table 5).

**Table 5:** Incidence rates for symptomatic and severe COVID-19 disease, using FDA and CEPI case definitions.

	FDA case definition		CEPI case definition	
	Virologically confirmed COVID-19	Severe COVID-19	Virologically confirmed COVID-19	Severe COVID-19
Population at risk	3670	3670	3670	3670
Number of RT-PCR positive cases	97	1	65	0
Cumulative incidence per 1000 participants [95% CI]	26.43 [21.48; 32.15]	0.27 [0.01; 1.52]	17.71 [13.70; 22.52]	0.00 [0.00; 1.00]
Total person-time at-risk (person-months)	41,410	42,308	41,653	42,322
Incidence rate per 1000 person-months [95% CI]	2.34 [1.90; 2.86]	0.02 [0.00; 0.13]	1.56 [1.20; 1.99]	0.00 [0.00; 0.00]

CI: confidence interval

#### COVID-19 symptoms

Most of the study population (88%) consistently reported symptoms throughout the study period every two weeks. Cough was the most common symptom reported, irrespective of RT-PCR result. Among participants who had a positive RT-PCR test result, loss of smell or taste was the second most common symptom. Participants with a negative RT-PCR test result were more likely to report one single symptom, the most common of which was cough, whereas those who tested positive commonly reported ≥2 symptoms. The most common symptom combination among RT-PCR-positive individuals was cough and loss of smell or taste and was generally of longer duration when compared to RT-PCR-negative subjects (S3 and S4 Figs).

### Asymptomatic SARS-CoV-2 infection

Of 260 infection-induced seroconversion events with self-reported symptom data available, 42 (16.2%) did not report any symptoms during the study period. There was no apparent trend in the percentages of asymptomatic infections relative to follow-up visits, except for the last visit (visit 6) where the highest percentage of asymptomatic infections was observed.

## Discussion

In this prospective cohort study, SARS-CoV-2 infections were assessed in HCWs in a large tertiary hospital in Mainz, Germany. The seroprevalence of anti-SARS-CoV-2 antibodies ranged from 2.7% to 4.3% prior to the rollout of COVID-19 vaccines, and from 1.3% to 6.2% after rollout. Seroconversion induced by natural infection could be distinguished from that induced by vaccination with or without natural infection by using the results from assays for antibodies against the N and S proteins, respectively. Infection-induced seroconversion rates showed a triphasic trend in SARS-CoV-2 infections, with an increase prior to the rollout of COVID-19 vaccines, followed by a decrease shortly after and then a sharp increase toward the end of the study. The overall cumulative incidence of COVID-19 disease in the study population was 26.4 per 1000 population, with three observed peaks in the incidence of positive RT-PCR test results: the first was around the time of the second wave of the pandemic, and the second and third peaks coincided with the periods when Delta and Omicron VOCs were dominant, respectively. In terms of clinical presentation, participants with positive RT-PCR results were more likely to report two or more symptoms, the most common of which were cough and smell or taste dysfunction. We estimated the proportion of asymptomatic infections at 16%.

### Seroprevalence and seroconversion

Data from observational studies in HCWs suggest that SARS-CoV-2 seroprevalence varies across settings [25]. This could reflect differences in study populations, background community transmission, dominant VOCs, COVID-19 vaccine uptake, criteria for screening, and adherence to personal protective equipment. A meta-analysis of studies conducted prior to the rollout of COVID-19 vaccines reported a pooled seroprevalence in HCWs of 8.7%, with the rates ranging from 4% in Asia to 12.7% in the US [31]. In Northern Germany, a prospective study reported a seroprevalence of anti-SARS-CoV-2 IgG antibodies that was comparable to that found in our study (4.4%) during the same period [32]; however, regions with a high background rate had a relatively higher seroprevalence, going up to 15.1% [33].

In the present study, a characteristic trend was observed in seroconversion rates relative to the COVID-19 vaccine rollout in early 2021. Prior to the COVID-19 vaccine rollout, there was a 1.6-fold increase in the seroconversion rate between September and December 2020. After the COVID-19 vaccine rollout, infection-specific seroconversion rates, as indicated by anti-N IgG antibodies, decreased while seroconversion suggesting vaccine-induced immunity (i.e., anti-S antibodies) increased up to the summer of 2021. This pattern reversed one year after the COVID-19 vaccine rollout, a period when the Delta and Omicron VOCs were dominant in Germany, with a five-fold sharp rise in infection-specific seroconversion while vaccine-induced anti-S antibodies fell 13-fold (from 88.6% to 6.8%) by the end of the study.

These findings suggest that although COVID-19 vaccines rolled out in the population in this study were efficacious, the duration of their immune protection was relatively short. A systematic literature review that assessed the kinetics of anti‐SARS‐CoV‐2 antibodies after the second dose of a primary cycle of COVID‐19 mRNA vaccination showed that the peak humoral response was reached at 21–28 days following the second dose, after which serum levels progressively fell by 55% to 85% four to six months after vaccination [34]. A longitudinal study in a large cohort of HCWs in Israel reported that anti-S SARS-CoV-2 IgG titers fell by a factor of 18.3 six months after receipt of the second vaccine dose of BNT162b2 COVID-19 vaccine [35]. The same study showed a strong correlation between anti-S IgG and neutralizing antibodies, which was dependent on the time since the second dose. Consistently, a significant waning of COVID-19 vaccine-induced immunity, as measured by vaccine effectiveness (VE), has been demonstrated throughout the pandemic. Two longitudinal studies of HCWs in the UK and Finland reported VE decreasing from about 85% to about 50% six months after dose two [36, 37]. A systematic literature review of 18 studies that were conducted during the Delta phase of the pandemic (i.e., June-December 2021) showed that irrespective of vaccine brand, VE against both infection and symptomatic disease decreased by about 20% to 30% after 6 months, although VE against severe disease remained high [38]. The peak infection rate toward the end of the present study, however, cannot be solely attributed to the waning of vaccine-induced immunity, given that Omicron, which has been shown to evade vaccine immunity, was the dominant circulating variant. Neutralization studies have demonstrated a reduction in neutralizing activity for Omicron in serum specimens obtained from recipients of two doses of BNT162b2 compared with neutralization activity against early pandemic viruses [39]. A recent study from the UK showed that, compared with the Delta variant, there was very limited VE against Omicron five months after the second dose of any vaccine [40]. The same study showed that although VE increased following booster doses, it then declined ten weeks after. Given the relatively low percentage of participants (76.5%) in the present study who reported receiving a COVID-19 vaccine booster compared with those who received the primary series (≥95%), the rise in infection rates toward the end of the study is not surprising. Nevertheless, the observed increase in infection rates in our population must be considered concerning. As these observations can be attributed at least partly to the reduced vaccine efficacy against the Omicron variant, our data fully support the global efforts made to develop Omicron-based vaccines [41].

### Virologically confirmed COVID-19 in the study population

The IR of virologically confirmed COVID-19 in the present study was estimated to be 2.3 per 1000 PMs. Nurses comprized most of the COVID-19 cases in our study compared with other occupational groups (OR 1.2; 95% CI: 0.8–1.8), with reported direct contact with confirmed COVID-19 cases. A systematic literature review and meta-analysis of 97 studies on HCWs during the first wave of the pandemic showed nurses were most at risk of COVID-19 infection [25]. Similarly, in a Scandinavian study, nurses had the highest risk of infection, and direct contact with COVID-19 patients was found to increase the risk for SARS-CoV-2 infection by more than three-fold [42].

Interestingly, the total number of 263 infection-specific seroconversions, which translates to a seroconversion rate of 6.6 per 1000 PMs, was 2.9 times higher than the estimated incidence of virologically confirmed COVID-19. Given that only 16% of those who seroconverted were asymptomatic, a significant number of symptomatic SARS-CoV-2 infections were likely missed despite the intense monitoring for symptoms throughout the study. The discrepancy between seroconversion rates and RT-PCR infection rates in the present study could be due to the fact that only participants reporting a predefined set of typical symptoms underwent RT-PCR testing, and thus, atypical presentations may have been missed. The largest percentage of symptomatic seroconversions missed by RT-PCR was among participants who seroconverted at the last follow-up visit (visit 6), suggesting that participants were less rigorous in reporting symptoms or there was a shift to atypical disease presentations with the Omicron variant. A large prospective study in the general population in the UK reported that during the Omicron prevalence, positive cases reported a shorter duration of symptoms, and they were 2–5 times less likely to report classic symptoms, including loss of taste or smell, compared with positive cases in the previous Delta-prevalent period [43]. The same study showed that sore throat was more likely to be reported in the Omicron period than in the Delta period. Sore throat was not included in the set of symptoms that triggered RT-PCR testing in our study. These findings indicate that for future non-interventional and interventional COVID-19 studies, screening for suspected COVID-19 episodes may need to be continuously modified according to fluctuations in reporting or changes in variant-specific clinical presentation.

Despite the lower-than-expected rate of RT-PCR positivity, the three peaks observed in the present study were consistent with both data on the background community transmission in the Rheinland-Pfalz (RP) region, where UM Mainz is located, as well as data on dominant variants in Germany (Fig 2). Pre-COVID-19-vaccine-rollout data indicated a ten-fold increase in the cumulative incidence of COVID-19 cases in the RP region, from 2.6 to 17.6 per 1000 population in September and December 2020, respectively [33]. These rates are consistent with those observed in the present study, suggesting that HCWs were at equal risk of infection as the general population. Importantly, only 14.4% of HCWs reported having direct contact with SARS-CoV-2–positive patients at enrollment. To prevent SARS-CoV-2 infections among the workforce of our hospital, thorough screening methods were introduced. All patients receiving inpatient treatment were tested for SARS-CoV-2 using RT-PCR at our hospital. Patients with a positive test result who required hospitalization were treated only in designated COVID-19 wards. If possible, treatment was postponed, and patients rescheduled until they had recovered from their infection. Similarly, patients receiving non-emergency outpatient treatment had to provide a negative SARS-CoV-2 RT-PCR test result before their consultation. SARS-CoV-2–positive patients were rescheduled if feasible. The observed equal risk of infection compared with the general population underlines that the screening methods introduced at our hospital did successfully reduce the exposure of our workforce. Following the COVID-19 vaccine rollout, the trends in RT-PCR positivity in the present study and the RP region appear to be consistent, apart from two notable differences. First, during April and May 2021, when the Alpha variant was dominant in Germany, the increase in cases in the RP region was not observed in our study, which showed a near-flat curve in the study population. This is likely to be the result of the early rollout of COVID-19 vaccines at UM Mainz. A meta-analysis of 18 studies that assessed post-vaccination SARS-CoV-2 infection during the Alpha phase estimated a ten-fold higher pooled incidence of COVID‐19 in unvaccinated HCWs compared with fully vaccinated HCWs [44]. In the present study, the peak uptake of COVID-19 vaccines was reached between May-July 2021, when the Alpha variant was dominant in Germany. In contrast, COVID-19 vaccine uptake in the general German population was relatively low during the same period (20%-40%) [45]. Second, although there was an increase in COVID-19 cases in the present study when the Omicron variant was dominant, the increase was lower than that observed in the RP region in the general population. This difference could be partly due to a relatively higher uptake of COVID-19 vaccine boosters in the study population. However, the low anti-S seropositivity rates at the end of the study suggest waning of vaccine immunity was more likely to have occurred following the earlier rollout of vaccination in the study population. Thus, other factors may have contributed to this difference, including undetected Omicron cases with atypical symptoms, differences in the population age structure, or the sustained implementation of infection-prevention measures in a tertiary-level healthcare setting. During our study, all HCWs had low-threshold access to SARS-CoV-2 testing, which may have led to early detection and control of new infections.

### Symptoms and severity of COVID-19

Most of the virologically confirmed COVID-19-positive episodes comprised two or more symptoms with varying combinations, which invariably included loss of smell or taste. A systematic literature review and meta-analysis of 97 studies in HCWs during the first wave of the pandemic showed anosmia, fever, and myalgia were likely to be associated with COVID-19 [25]. In contrast, individuals with COVID-19–negative episodes in the present study were more likely to report single symptoms with relatively short durations, the most common of which was cough. Although the results in this study suggest that active case finding based on smell or taste dysfunction and cough would help detect most symptomatic SARS-CoV-2 infections, variant-specific clinical presentations may shift to milder and/or atypical symptoms, as it was the case with the Omicron variant [43].

Only one of the COVID-19 cases in our study met the case definition of severe disease (and this participant (1/97; 1%) was hospitalized). The very low proportion of severe cases in the present study population may be related to the absence of risk factors for severe COVID-19 disease. In a systematic literature review, a high prevalence of comorbidities in HCWs (18.4%) explained a fifteen-fold higher prevalence of hospitalizations for COVID-19 (approx. 15%) [46], than that of our study. In contrast, in the present study population, only 2.6% of subjects (94/3670) reported having comorbidities. Additionally, the present study was carried out in a population with early uptake of COVID-19 vaccines, which were demonstrated to provide high protection against severe disease–irrespective of circulating variants [38].

Only 16% of the HCWs who seroconverted in our study did not report any symptoms. A systematic literature review and meta-analysis of 77 studies that were conducted up to February 2021 estimated the pooled percentage of asymptomatic infections in HCWs to be 30% (95% CI: 21.1%-38.9%) [47]. The prospective nature of our study, whereby participants were invited to report symptoms continuously, may have resulted in a higher detection rate of symptomatic infection. In Spain, two cross-sectional studies reported that between 39% and 49% of seropositive HCWs were asymptomatic, although in the latter study, almost half of the seropositive HCWs recalled minor symptoms when specifically asked [48, 49]. In contrast, a national, multi-center, prospective study of Belgian HCWs during the first wave of the pandemic (April to December 2020) reported the proportion of asymptomatic SARS-CoV-2 infections to be 12% [50]. Concerns about introducing infection into the hospital setting, combined with easy access to testing, are also likely to have motivated higher reporting rates of symptoms. Although our low-threshold active surveillance is more sensitive for detecting asymptomatic infections, this approach is resource intensive and may not be suitable in most settings. For example, we had to test about 34 participants with RT-PCR to detect one COVID-19-positive participant.

In addition to providing epidemiological information on SARS-CoV-2 infections in the study population, the present study’s purpose was to facilitate the enrollment of participants into a phase III clinical vaccine trial. Thus, the protocol was amended to maximize methodological similarity with the trial, including extending the study duration and adjusting the content and timing of data collection, e.g., the frequency of self-reported symptoms, RT-PCR testing algorithm for suspected cases, and COVID-19 vaccination data. However, the relatively low SARS-CoV-2 infection rates among the participants hindered any formal efficacy analyses in the trial, and data from our study were not used as an external control group.

### Study limitations

Our study has some limitations. First, the sampling method was convenience-based. However, given the very high participation rate in this study (approximately 40% of all HCWs at UM Mainz), the study population may not be representative of the overall HCW population at UM Mainz. Second, the study relied on self-reporting of symptoms, which may have resulted in recall bias or underreporting. However, the frequency of the reporting and the high awareness of COVID-19 disease probably reduced the risk of recall bias. To reduce underreporting of symptoms, the study team received automated alerts for participants who had multiple missing reports. Our analysis of missing data also suggested that most of the participants were compliant with reporting COVID-19 symptoms throughout the study, with only 3% of the participants missing the reporting of symptoms on three or more occasions. Third, the study lost 33.5% of its population, primarily due to discontinuation in our study to enroll in a clinical trial assessing an investigational COVID-19 vaccine. We decided against double reporting in those subjects to limit the burden on the individual participant and to ensure that the here presented data are not confounded by placebo or unapproved vaccines. The remaining participants, who were lost to follow-up or withdrew, account for 8,7% of the study population. This seems not exceedingly high in a study period of 19 months. Although selection bias cannot be fully ruled out, the high COVID-19 vaccination coverage among those who remained in our study suggests similar attitudes toward vaccination to those who discontinued. Fourth, this study was not designed to assess the potential impact of personal protective equipment, which is relevant given that most COVID-19 cases in our study reported direct contact with confirmed COVID-19 patients. Last, this study was conducted in a high-risk population over a relatively long period, when investigational COVID-19 vaccines were rolled out in a setting of changing circulating SARS-CoV-2 variants, including the highly transmissible Omicron variant. Thus, the findings should be interpreted with caution, given the complex interactions between the host, SARS-CoV-2 variants, and the environment.

## Conclusion

In conclusion, a large sample of HCWs from Mainz was successfully followed up for both symptomatic and asymptomatic SARS-CoV-2 infections. Infection rates were found to be in line with or even lower than those observed in the general population, despite the intense follow-up and high exposure rates associated with the professions of the participants. Following the introduction of COVID-19 vaccination, infection rates were markedly lower than those in the general population, despite waning antibody levels. This emphasizes the importance of maintaining non-pharmaceutical interventions in the healthcare setting, particularly in the context of highly transmissible variants. The rates of asymptomatic infections were relatively low in our prospective study, which suggests that high detection rates can be achieved with systematic screening and easily accessible testing. With the rapidly evolving epidemiology of SARS-CoV-2, continued monitoring of infection rates in at-risk populations is essential. Due to the early rollout of COVID-19 vaccines under emergency-use authorization at the study site, the secondary goal of the present study, to provide an external control group for the efficacy analyses in a parallel clinical trial, was only partially achieved. Even though the study procedures were successfully aligned to the interventional trial and many study participants successfully enrolled in the trial, the observed COVID-19 disease rates in both this study and the trial were too low to enable a meaningful vaccine efficacy analysis. The concept, nevertheless, warrants consideration in future studies, as the study demonstrated the feasibility of a rapid adaptation of observational studies in public health emergencies to support the clinical development of lifesaving interventions.

## Supporting information

S1 Appendix Study team and administrative structure. (DOCX)

S2 Appendix Questionnaires. (DOCX)

S1 Fig SARS-CoV-2 RT-PCR testing algorithm. (DOCX)

S2 Fig Study enrollment and timing of discontinuation. (DOCX)

S3 Fig Combination of symptoms reported by suspected COVID-19 cases. (DOCX)

S4 Fig Duration of clinical symptoms of suspected COVID-19 cases. (DOCX)

S1 Table Case definitions. (DOCX)

S2 Table Schedule of visits and number of subjects in each visit. (DOCX)

S3 Table Data collection and timing of data collection. (DOCX)

S4 Table Summary of antibody testing. (DOCX)

S5 Table Baseline characteristics by serostatus. (DOCX)

S6 Table Reasons for discontinuation. (DOCX)

S7 Table Baseline characteristics, including demographics and COVID-19 disease risk factors, among healthcare workers at Universitätsmedizin Mainz, Germany, stratified by vaccination status. (DOCX)

S8 Table Crude and adjusted seroprevalence of anti-SARS-CoV-2 antibodies at scheduled visits 3–5, stratified by age/sex/comorbidities and vaccine uptake. (DOCX)

## References

[1] Zhu N, Zhang D, Wang W, Li X, Yang B, Song J, et al. A Novel Coronavirus from Patients with Pneumonia in China, 2019. N Engl J Med. 2020;382(8):727–33. doi: 10.1056/NEJMoa2001017 31978945 PMC7092803

[2] The World Health Organization [Internet]. Coronavirus disease 2019 (COVID-19) Situation Reports 11, 22, and 52. 2020 [cited 2022 Oct 25]. Available from: https://www.who.int/emergencies/diseases/novel-coronavirus-2019/situation-reports

[3] The World Health Organization [Internet]. WHO Coronavirus Disease (COVID-19) Dashboard: WHO. 2021 [cited 2022 Oct 25]. Available from: https://covid19.who.int

[4] The World Health Organisation [Internet]. Tracking SARS-CoV-2 variants. 2022 [cited 2022 Oct 25]. Available from: https://www.who.int/activities/tracking-SARS-CoV-2-variants

[5] Lin L, Liu Y, Tang X, He D. The Disease Severity and Clinical Outcomes of the SARS-CoV-2 Variants of Concern. Front Public Health. 2021;9:775224. doi: 10.3389/fpubh.2021.775224 34917580 PMC8669511

[6] Nyberg T, Ferguson NM, Nash SG, Webster HH, Flaxman S, Andrews N, et al. Comparative analysis of the risks of hospitalisation and death associated with SARS-CoV-2 omicron (B.1.1.529) and delta (B.1.617.2) variants in England: a cohort study. Lancet. 2022;399(10332):1303–12. doi: 10.1016/S0140-6736(22)00462-7 35305296 PMC8926413

[7] Robert Koch-Institut: COVID-19-Dashboard 2022 [Internet]. 2022 [cited 2022 Oct 25]. Available from: https://experience.arcgis.com/experience/478220a4c454480e823b17327b2bf1d4

[8] Hale T, Angrist N, Goldszmidt R, Kira B, Petherick A, Phillips T, et al. A global panel database of pandemic policies (Oxford COVID-19 Government Response Tracker). Nat Hum Behav. 2021;5(4):529–38. doi: 10.1038/s41562-021-01079-8 33686204

[9] World Health Organisation. Therapeutics and COVID-19: Living guideline, 13 January 2023 [Internet]. 2023 [cited 2022 Oct 25]. Available from: https://www.who.int/publications/i/item/WHO-2019-nCoV-therapeutics-2023.1

[10] Wang K, Wang L, Li M, Xie B, He L, Wang M, et al. Real-Word Effectiveness of Global COVID-19 Vaccines Against SARS-CoV-2 Variants: A Systematic Review and Meta-Analysis. Front Med (Lausanne). 2022;9:820544. doi: 10.3389/fmed.2022.820544 35665358 PMC9160927

[11] World Health Organisation [Internet]. Interim statement on decision-making considerations for the use of variant updated COVID-19 vaccines (17 June 2022). 2022 [cited 2022 Oct 25]. Available from: https://www.who.int/news/item/17-06-2022-interim-statement-on-decision-making-considerations-for-the-use-of-variant-updated-covid-19-vaccines

[12] VIPER Group COVID-19 Vaccine Tracker Team [Internet]. COVID-19 Vaccine Tracker. 2022 [cited 2022 Oct 25]. Available from: https://covid19.trackvaccines.org/

[13] German Ministry of Health [Internet]. COVID-19 vaccines tracker. 2022 [cited 2022 Oct 25]. Available from: https://impfdashboard.de/

[14] Yang J, Zheng Y, Gou X, Pu K, Chen Z, Guo Q, et al. Prevalence of comorbidities and its effects in patients infected with SARS-CoV-2: a systematic review and meta-analysis. Int J Infect Dis. 2020;94:91–5. doi: 10.1016/j.ijid.2020.03.017 32173574 PMC7194638

[15] Takagi H. Risk and protective factors of SARS-CoV-2 infection. J Med Virol. 2021;93(2):649–51. doi: 10.1002/jmv.26427 32790191 PMC7436481

[16] Hariyanto TI, Kurniawan A. Dyslipidemia is associated with severe coronavirus disease 2019 (COVID-19) infection. Diabetes Metab Syndr. 2020;14(5):1463–5. doi: 10.1016/j.dsx.2020.07.054 32771919 PMC7395301

[17] Li R, Tian J, Yang F, Lv L, Yu J, Sun G, et al. Clinical characteristics of 225 patients with COVID-19 in a tertiary Hospital near Wuhan, China. J Clin Virol. 2020;127:104363. doi: 10.1016/j.jcv.2020.104363 32298988 PMC7194914

[18] Zhu Z, Hasegawa K, Ma B, Fujiogi M, Camargo CA Jr., Liang L. Association of asthma and its genetic predisposition with the risk of severe COVID-19. J Allergy Clin Immunol. 2020;146(2):327–9 e4. 32522462 10.1016/j.jaci.2020.06.001 PMC7423602

[19] Arentz M, Yim E, Klaff L, Lokhandwala S, Riedo FX, Chong M, et al. Characteristics and Outcomes of 21 Critically Ill Patients With COVID-19 in Washington State. JAMA. 2020;323(16):1612–4. doi: 10.1001/jama.2020.4326 32191259 PMC7082763

[20] Zhang Q, Bastard P, Liu Z, Le Pen J, Moncada-Velez M, Chen J, et al. Inborn errors of type I IFN immunity in patients with life-threatening COVID-19. Science. 2020;370(6515). 32972995 10.1126/science.abd4570 PMC7857407

[21] Chen W, Tian Y, Li Z, Zhu J, Wei T, Lei J. Potential Interaction Between SARS-CoV-2 and Thyroid: A Review. Endocrinology. 2021;162 (3). doi: 10.1210/endocr/bqab004 33543236 PMC7953946

[22] Su S, Chen R, Zhang S, Shu H, Luo J. Immune system changes in those with hypertension when infected with SARS-CoV-2. Cell Immunol. 2022;378:104562. doi: 10.1016/j.cellimm.2022.104562 35901625 PMC9183242

[23] Nguyen LH, Drew DA, Graham MS, Joshi AD, Guo CG, Ma W, et al. Risk of COVID-19 among front-line health-care workers and the general community: a prospective cohort study. Lancet Public Health. 2020;5(9):e475–e83. doi: 10.1016/S2468-2667(20)30164-X 32745512 PMC7491202

[24] Iversen K, Bundgaard H, Hasselbalch RB, Kristensen JH, Nielsen PB, Pries-Heje M, et al. Risk of COVID-19 in health-care workers in Denmark: an observational cohort study. Lancet Infect Dis. 2020;20(12):1401–8. doi: 10.1016/S1473-3099(20)30589-2 32758438 PMC7398038

[25] Gomez-Ochoa SA, Franco OH, Rojas LZ, Raguindin PF, Roa-Diaz ZM, Wyssmann BM, et al. COVID-19 in Health-Care Workers: A Living Systematic Review and Meta-Analysis of Prevalence, Risk Factors, Clinical Characteristics, and Outcomes. Am J Epidemiol. 2021;190(1):161– doi: 10.1093/aje/kwaa191 32870978 PMC7499478

[26] Verger P, Scronias D, Dauby N, Adedzi KA, Gobert C, Bergeat M, et al. Attitudes of healthcare workers towards COVID-19 vaccination: a survey in France and French-speaking parts of Belgium and Canada, 2020. Euro Surveill. 2021;26(3). doi: 10.2807/1560-7917.ES.2021.26.3.2002047 33478623 PMC7848677

[27] Clopper CJ, Pearson ES. The use of confidence or fiducial limits illustrated in the case of the binomial. Biometrika. 1934;26(4):404–13.

[28] Speybroeck N, Devleesschauwer B, Joseph L, Berkvens D. Misclassification errors in prevalence estimation: Bayesian handling with care. International journal of public health. 2013;58(5):791–5. 23263198 10.1007/s00038-012-0439-9

[29] Garwood F. Fiducial limits for the Poisson distribution. Biometrika. 1936;28(3–4):437–42.

[30] RStudio Team. RStudio: Integrated Development for R [Internet]. Boston (MA): RStudio, PBC; 2020 [cited 2022 Aug 23]. Available from: http://www.rstudio.com/.

[31] Galanis P, Vraka I, Fragkou D, Bilali A, Kaitelidou D. Seroprevalence of SARS-CoV-2 antibodies and associated factors in healthcare workers: a systematic review and meta-analysis. J Hosp Infect. 2021;108:120–34. doi: 10.1016/j.jhin.2020.11.008 33212126 PMC7668234

[32] Herzberg J, Vollmer T, Fischer B, Becher H, Becker AK, Sahly H, et al. Prospective Sero-epidemiological Evaluation of SARS-CoV-2 among Health Care Workers in a German Secondary Care Hospital. Int J Infect Dis. 2021;102:136–43. doi: 10.1016/j.ijid.2020.10.026 33075538 PMC7566663

[33] Finkenzeller T, Faltlhauser A, Dietl KH, Paetzel C, Szczypien N, Klawonn F, et al. [SARS-CoV-2 antibodies in ICU and clinic staff: From Germany’s region with the highest infection rate]. Med Klin Intensivmed Notfmed. 2020;115(Suppl 3):139–45. 33274410 10.1007/s00063-020-00761-5 PMC7713905

[34] Notarte KI, Guerrero-Arguero I, Velasco JV, Ver AT, Santos de Oliveira MH, Catahay JA, et al. Characterization of the significant decline in humoral immune response six months post-SARS-CoV-2 mRNA vaccination: A systematic review. J Med Virol. 2022;94(7):2939–61. doi: 10.1002/jmv.27688 35229324 PMC9088566

[35] Levin EG, Lustig Y, Cohen C, Fluss R, Indenbaum V, Amit S, et al. Waning Immune Humoral Response to BNT162b2 Covid-19 Vaccine over 6 Months. N Engl J Med. 2021;385(24):e84. doi: 10.1056/NEJMoa2114583 34614326 PMC8522797

[36] Bedston S, Akbari A, Jarvis CI, Lowthian E, Torabi F, North L, et al. COVID-19 vaccine uptake, effectiveness, and waning in 82,959 health care workers: A national prospective cohort study in Wales. Vaccine. 2022;40(8):1180–9. doi: 10.1016/j.vaccine.2021.11.061 35042645 PMC8760602

[37] Poukka E, Baum U, Palmu AA, Lehtonen TO, Salo H, Nohynek H, et al. Cohort study of Covid-19 vaccine effectiveness among healthcare workers in Finland, December 2020—October 2021. Vaccine. 2022;40(5):701–5. doi: 10.1016/j.vaccine.2021.12.032 34953607 PMC8683266

[38] Feikin DR, Higdon MM, Abu-Raddad LJ, Andrews N, Araos R, Goldberg Y, et al. Duration of effectiveness of vaccines against SARS-CoV-2 infection and COVID-19 disease: results of a systematic review and meta-regression. Lancet. 2022;399(10328):924–44. doi: 10.1016/S0140-6736(22)00152-0 35202601 PMC8863502

[39] Cele S, Jackson L, Khoury DS, Khan K, Moyo-Gwete T, Tegally H, et al. SARS-CoV-2 Omicron has extensive but incomplete escape of Pfizer BNT162b2 elicited neutralization and requires ACE2 for infection. medRxiv. 2021. doi: 10.1101/2021.12.08.21267417 34909788 PMC8669855

[40] Andrews N, Stowe J, Kirsebom F, Toffa S, Rickeard T, Gallagher E, et al. Covid-19 Vaccine Effectiveness against the Omicron (B.1.1.529) Variant. N Engl J Med. 2022;386(16):1532–46. doi: 10.1056/NEJMoa2119451 35249272 PMC8908811

[41] Viveiros-Rosa SG, Mendes CDS, Farfán-Cano GG, El-Shazly M. The race for clinical trials on Omicron-based COVID-19 vaccine candidates: Updates from global databases. Narra J 2022;2(3):e88. 10.52225/narra.v2i3.88 PMC10914133 38449904

[42] Rudberg AS, Havervall S, Månberg A, Jernbom Falk A, Aguilera K, Ng H, et al. SARS-CoV-2 exposure, symptoms and seroprevalence in healthcare workers in Sweden. Nat Commun. 2020;11(1):5064. doi: 10.1038/s41467-020-18848-0 33033249 PMC7544689

[43] Menni C, Valdes AM, Polidori L, Antonelli M, Penamakuri S, Nogal A, et al. Symptom prevalence, duration, and risk of hospital admission in individuals infected with SARS-CoV-2 during periods of omicron and delta variant dominance: a prospective observational study from the ZOE COVID Study. Lancet. 2022;399(10335):1618–24. doi: 10.1016/S0140-6736(22)00327-0 35397851 PMC8989396

[44] Chandan S, Khan SR, Deliwala S, Mohan BP, Ramai D, Chandan OC, et al. Postvaccination SARS-CoV-2 infection among healthcare workers: A systematic review and meta-analysis. J Med Virol. 2022;94(4):1428–41. doi: 10.1002/jmv.27457 34783055 PMC8661690

[45] European Centre for Disease Prevention and Control [Internet]. COVID-19 vaccines tracker [cited 2022 Oct 25]. Available from: https://vaccinetracker.ecdc.europa.eu/public/extensions/covid-19/vaccine-tracker.html#uptake-tab

[46] Gholami M, Fawad I, Shadan S, Rowaiee R, Ghanem H, Hassan Khamis A, et al. COVID-19 and healthcare workers: A systematic review and meta-analysis. Int J Infect Dis. 2021;104:335–46. doi: 10.1016/j.ijid.2021.01.013 33444754 PMC7798435

[47] Ma Q, Liu J, Liu Q, Kang L, Liu R, Jing W, et al. Global Percentage of Asymptomatic SARS-CoV-2 Infections Among the Tested Population and Individuals With Confirmed COVID-19 Diagnosis: A Systematic Review and Meta-analysis. JAMA Network Open. 2021;4(12):e2137257–e. doi: 10.1001/jamanetworkopen.2021.37257 34905008 PMC8672238

[48] Garralda Fernandez J, Molero Vilches I, Bermejo Rodríguez A, Cano Torres I, Colino Romay EI, García Arata I, et al. Impact of SARS-CoV-2 pandemic among health care workers in a secondary teaching hospital in Spain. PLoS One. 2021;16(1):e0245001. doi: 10.1371/journal.pone.0245001 33444392 PMC7808590

[49] Galán MI, Velasco M, Casas ML, Goyanes MJ, Rodríguez-Caravaca G, Losa-García JE, et al. Hospital-Wide SARS-CoV-2 seroprevalence in health care workers in a Spanish teaching hospital. Enferm Infecc Microbiol Clin. 2020;40(6):302–9. 10.1016/j.eimc.2020.11.015 PMC7833995 33485676

[50] Mortgat L, Verdonck K, Hutse V, Thomas I, Barbezange C, Heyndrickx L, et al. Prevalence and incidence of anti-SARS-CoV-2 antibodies among healthcare workers in Belgian hospitals before vaccination: a prospective cohort study. BMJ Open. 2021;11(6):e050824. doi: 10.1136/bmjopen-2021-050824 34187832 PMC8245288

