# Supplementary material for: A prospective cohort study of SARS-CoV-2 infection-induced seroconversion and disease incidence in German healthcare workers before and during the rollout of COVID-19 vaccines
Source: PLoS One. 2024 Jan 30;19(1):e0294025. doi: 10.1371/journal.pone.0294025 (PMC10826949; doi:10.1371/journal.pone.0294025)
Supplement: S1 Appendix — (DOCX) [file pone.0294025.s001.docx]

# Study team

**Investigators**: Andrea Nebgen, Winter, Julia, Philip Koliopoulos, and Yildiz Gökdemir

**Study nurses**: Sabine Wiegert, Melanie Kleinhanß, Sabine Lindau, Nina Nitschke, Suna Hach, and Mirjam Larisika

**Study Coordination**: Laura Harder, Markus Heymanns, and Katrin Hinz

**Quality assurance/data management**: Alexandra Dieter, Rahel Irina Huissel, Lisa Knipfer, Julia Popow, and Daniel Volk

**Laboratory support**: Sebastian Wirsching, Claudius Meyer, Katja Hilbert, Britta Gröhndal, Stefan Runkel, and Walter Hitzler

**Scientific support**: Irene Krämer and Christoph Düber

# Administrative structure

| Role | Responsibilities | Key Responsible Person(s)/Entity |
| --- | --- | --- |
| **Funding institution** | - Funding and oversight | Cornelia Oostvogels, SVP Area Head Infectious Diseases  CureVac SE  Schumannstraße 27  60325 Frankfurt  Germany |
| **Sponsor** | - Protocol development and amendments - Registration in EU PAS register - Coordination between and communication with CureVac SE, study site and monitors - Epidemiological support - Data management - Data quality - Statistical analyses - Communication with Ethics Committee - Reporting | Thomas Verstraeten, MD, MSc  Managing Director  P95 Epidemiology and Pharmacovigilance Koning Leopold III laan 1 3001 Heverlee Belgium |
| **Investigator & study site** | - Protocol and protocol amendment review - Study conduct - Communication with sponsor | Principal Investigator:  Univ. Prof. Dr. med. Stephan Gehring Co-Investigator:  Dr. med. Frank Kowalzik  Universitätsmedizin Mainz (UM Mainz; University Medical Center of the Johannes Gutenberg University Mainz) Zentrum für Kinder- und Jugendmedizin Langenbeckstraße 1 55131 Mainz Germany |
| **Data management at study site** | - Electronic data capture and database - Data management - Data quality - Data transfer to sponsor | Tobias Engelmann Interdisziplinäres Zentrum Klinische Studien (IZKS)  UM Mainz  Langenbeckstraße 1 55131 Mainz Germany |
| **Monitoring** | - Quality assurance | Harmony Clinical Research bv Brusselsesteenweg 159 9090 Melle Belgium |
