# Supplementary material for: A prospective cohort study of SARS-CoV-2 infection-induced seroconversion and disease incidence in German healthcare workers before and during the rollout of COVID-19 vaccines
Source: PLoS One. 2024 Jan 30;19(1):e0294025. doi: 10.1371/journal.pone.0294025 (PMC10826949; doi:10.1371/journal.pone.0294025)
Supplement: S2 Appendix — (DOCX) [file pone.0294025.s002.docx]

# Questionnaire at enrollment

| Contact information (Auto-generated post-user authentication) | | |
| --- | --- | --- |
| 1 | First name |  |
| 2 | Last name |  |
| 3 | Personnel number |  |
| 4 | Mobile phone number |  |
| 5 | Pseudonymisation number |  |
| 6 | Date | _ _-_ _-_ _ _ _ |
| Personal information | | |
| 7 | Gender | ( ) Female ( ) Male ( ) Divers |
| 8 | Date of birth | _ _-_ _-_ _ _ _ |
| Work information | | |
| 9 | Occupational group | ( ) Doctor ( ) Care ( ) Student ( ) Other |
| 10 | Own clinic/department |  |
| 11 | Building number | _ _ _ |
| 12 | Direct care of SARS-CoV-2 patient | ( ) Yes ( ) No |
| Travel and contact information | | |
| 13 | Have you possibly been in a SARS-CoV-2 risk area? | ( ) Yes ( ) No |
| 14 | Did you have aware contact with a SARS-CoV-2 positive or probably positive person? | ( ) Yes ( ) No |
| Diagnostics | | |
| 15, 16, 17 | Have you already had a diagnostic procedure (swab/follow-up test) independent from a study? | ( ) Yes ( ) No |
|  | If yes, which? | ( ) Swab ( ) Follow-up test  ( ) Blood test ( ) Other |
|  | If yes, when? (Date) | _ _-_ _-_ _ _ _ |
| Risk information | | |
| 18 | Smoker | ( ) Yes ( ) No |
| 19 | Disorders of airways and lung | ( ) Yes ( ) No |
| 20 | Disorders of the cardiovascular system | ( ) Yes ( ) No |
| 21 | Immune weakness | ( ) Yes ( ) No |

# Questionnaire at follow-up visits

| Contact information (Auto-generated post-user authentication) | | |
| --- | --- | --- |
| 1 | First name |  |
| 2 | Last name |  |
| 3 | Personnel number |  |
| 4 | Mobile phone number |  |
| 5 | Pseudonymisation number |  |
| 6 | Date | _ _-_ _-_ _ _ _ |
| Occupational, contact, travel, and other risk information | | |
| 1.a | What is your current job title *(original German response options)* | ( ) Arzt/Ärztin  ( ) Krankenschwester/Krankenpfleger  ( ) Krankenpflegehelfer/-helferin  ( ) Hebamme  ( ) Medizinische/r Fachangestellte/r  ( ) Medizinisch-technische/r Radiologieassistent/in  ( ) Operationstechnische/r Assistent/in  ( ) Laborpersonal  ( ) Humanmedizin/Zahnmedizinstudent  ( ) Notfallsanitäter/in oder Rettungsassistent/in  ( ) Verwaltungspersonal  ( ) Sonstige |
| 1.b | If other, specify | ……….. |
| 2 | In your current work, do you have any direct contact with patients? | ( ) Yes ( ) No |
| If yes, proceed to question 3. If not, skip to question 6 | | |
| 3 | Over the past 6 weeks, what is the average hours per day where you had direct contact with patients? | …..hours/day |
| 4 | Did you have direct contact with patients admitted for/with SARS-COV-2 infection? | ( ) Yes ( ) No ( ) Not sure |
| 5 | Did you work at a specific COVID-19 ward? | ( ) Yes ( ) No |
| 6, 7 | Did you travel to another country in the last 6 weeks? | ( ) Yes ( ) No |
|  | If yes, list the countries and number of days spent | Country ___________ # Days _ _  Country ___________ # Days _ _  Country ___________ # Days _ _ |
| 8 | Did you have contact with a confirmed or suspected SARS-COV-2 infected **person**, including your households (if any)? | ( ) Yes ( ) No ( ) Not sure |
| 9 | Which of the following applies to your habits during the last 6 weeks? | ( ) I started smoking  ( ) I quit smoking  ( ) I smoke  ( ) I don´t smoke |
| 10 | Have you had lung disease diagnosed in you during the last 6 weeks? | ( ) Yes  ( ) No |
| 11 | Have you had cardiovascular disease diagnosed in you during the last 6 weeks? | ( ) Yes  ( ) No |
| 12 | Have you had immune suppression diagnosed in you during the last 6 weeks, or are you taking immunosuppresssive medicines? | ( ) Yes  ( ) No |
| SARS-Cov-2 diagnosis | | |
| 13, 14, 15, 16, 17 | Have you been diagnosed with a SARS-COV-2 infection in the last 6 weeks? | ( ) Yes ( ) No ( ) Not sure |
|  | Was an upper respiratory swab taken? | ( ) Yes ( ) No |
|  | If yes, when was the sample taken? (Date) | _ _-_ _-_ _ _ _ |
|  | Was a blood sample taken? | ( ) Yes ( ) No |
|  | If yes, when was the sample taken? (Date) | _ _-_ _-_ _ _ _ |

# Questionnaire for daily symptom checks

| Contact information (Auto-generated post-user authentication) | | | |
| --- | --- | --- | --- |
| 1 | First name |  | |
| 2 | Last name |  | |
| 3 | Personnel number |  | |
| 4 | Mobile phone number |  | |
| 5 | Pseudonymisation number (to be filled out by app) |  | |
| 6 | Date (to be filled out by app) | _ _-_ _-_ _ _ _ | |
| Daily symptoms check | | |  |
| 7 | Which of the following symptoms did you experience in the last 24 hours? | ( ) cough (with or without sputum)  ( ) shortness of breath  ( ) chills (shivers)  ( ) smell or taste dysfunction  ( ) diarrhea | ( ) Yes ( ) No |
| 8 | Did you measure your temperature? | ( ) Yes ( ) No |  |
|  | If yes, when did you measure your temperature? | _ _:_ _ (Time) |  |
|  | If yes, how high was your body temperature? | ……° C |  |

# Questionnaire for confirmed COVID-19 cases

| Contact information | | |  |
| --- | --- | --- | --- |
| 1 | First name |  |  |
| 2 | Last name |  |  |
| 3 | Personnel number |  |  |
| 4 | Mobile phone number |  |  |
| 4 | Pseudonymisation number |  |  |
| 6 | Date | _ _-_ _-_ _ _ _ |  |
| 7 | Investigator/Interviewer initials |  |  |
| COVID-19 symptoms | | | |
| 8, 9, 10 | Which of the following symptoms did you experience in the last 2 weeks? | ( ) cough (with or without sputum)  ( ) shortness of breath  ( ) fever ≥37.8°C  ( ) chills  ( ) fatigue (extreme tiredness)/malaise (feeling unwell)  ( ) confusion  ( ) muscle aches  ( ) nasal obstruction  ( ) runny nose  ( ) sore throat  ( ) headache  ( ) diarrhea  ( ) nausea or vomiting  ( ) smell or taste dysfunction  ( ) abdominal pain | |
|  | If any symptom(s), state when the first symptom(s) appeared (date) | _ _-_ _ -_ _ _ _ | |
|  | If any symptom(s), state when the symptom(s) resolved (date) | _ _-_ _-_ _ _ _ | |
| Travel, contact and other risk information | | |  |
| 11, 12 | Did you travel to another country in the last 6 weeks? | ( ) Yes ( ) No |  |
|  | If yes, list the countries and number of days spent | Country ___________ # Days _ _  Country ___________ # Days _ _  Country ___________ # Days _ _ |  |
| 13 | Did you have contact with a confirmed or suspected SARS-CoV-2 infected person? | ( ) Yes ( ) No ( ) Not sure |  |
| 14 | Which of the following applies to your habits during the last 6 weeks? | ( ) I started smoking  ( ) I quit smoking  ( ) I don´t smoke  ( ) I smoke |  |
| 15 | Have you had lung disease diagnosed in you during the last 6 weeks? | ( ) Yes  ( ) No |  |
| 16 | Have you had cardiovascular disease diagnosed in you during the last 6 weeks? | ( ) Yes  ( ) No |  |
| 17 | Have you had immune suppression diagnosed in you during the last 6 weeks, or are you taking immunosuppresssive medicines? | ( ) Yes  ( ) No |  |
|  |  |  |  |
|  |  |  |  |

| Clinical course: complications | | |
| --- | --- | --- |
| 18 | Pneumonia | ( ) Yes ( ) No ( ) Unknown |
| 19 | Radiological confirmation of pneumonia | ( ) Yes ( ) No ( ) Unknown  ( ) Yes, CT  ( ) Yes, Other (specify): |
| 20 | ARDS (Acute Respiratory Distress Syndrome) | ( ) Yes ( ) No ( ) Unknown |
| 21, 22, 23 | Hospitalisation | ( ) Yes ( ) No ( ) Unknown |
|  | If yes, hospital admission date | _ _-_ _-_ _ _ _ |
|  | If yes, hospital discharge date | _ _-_ _-_ _ _ _ |
| 24, 25 | Other healthcare interaction (Emergency Room, Primary Health Care) | ( ) Yes ( ) No ( ) Unknown |
|  | If yes, specify date | _ _-_ _-_ _ _ _ |
| 26, 27, 28 | ICU (Intensive Care Unit) admission | ( ) Yes ( ) No ( ) Unknown |
|  | If yes, ICU admission date | _ _-_ _-_ _ _ _ |
|  | If yes, ICU discharge date | _ _-_ _-_ _ _ _ |
| 29, 30 | Mechanical ventilation (MV) | ( ) Yes ( ) No ( ) Unknown |
|  | If yes, number of days on MV |  |
| 31 | Extracorporeal membrane oxygenation (CMO) | ( ) Yes ( ) No ( ) Unknown |
| 32 | Acute renal failure | ( ) Yes ( ) No ( ) Unknown |
| 33 | Acute liver failure | ( ) Yes ( ) No ( ) Unknown |
| 34 | Acute neurologic dysfunction | ( ) Yes ( ) No ( ) Unknown |
| 35 | Cardiac failure | ( ) Yes ( ) No ( ) Unknown |
| 36 | Hypotension requiring vasopressors | ( ) Yes ( ) No ( ) Unknown |
| 37 | Consumptive coagulopathy | ( ) Yes ( ) No ( ) Unknown |
| Clinical assessment  *Score according to the NEWS2 scoring system. In case of inpatient treatment, the worst score should be documented for each day of inpatient treatment.* | | |
| 38 | Respiration rate (rr/min) | …...; Worst score: |
| 39 | SpO_2_ (%) | ……; Worst score: |
| 40 | PaO_2_/FiO_2_ (mmHg)  (in severe cases and/or inpatients) | ……; Worst score: |
| 41 | Systolic blood pressure (mmHg) | ……; Worst score: |
| 42 | Diastolic blood pressure (mmHg) | ……; Worst score: |
| 43 | Pulse (bpm) | ……; Worst score: |
| 44 | Consciousness (alert or CVPU) | …….; Worst score: |
| Clinical course : outcome | | |
| 45, 46, 47, 48, 49 | Did the subject die during follow-up? | ( ) Yes ( ) No ( ) Unknown |
|  | If died, date of death | _ _-_ _-_ _ _ _ |
|  | If died, cause of death related to COVID-19? | ( ) Yes ( ) No ( ) Unknown |
|  | If died, was postmortem performed? | ( ) Yes ( ) No ( ) Unknown |
|  | If postmortem was performed, what was the result? |  |
| Lab tests/investigations not performed as part of the study | | |
| 50, 51 | Molecular (RT-PCR) | ( ) Yes ( ) No ( ) Unknown |
|  | If yes, date of the first PCR | _ _-_ _-_ _ _ _ |
| 52, 53, 54, 55 | If other molecular tests were performed (whole/partial genome sequencing, other), please specify |  |
|  | If other molecular tests were performed, which type of specimen (OroPharyngeal/NasoPharyngeal swabs, sputum)? | ( ) OP ( ) NP ( ) Sputum |
|  | If other molecular tests were performed, specify the date (for each type of specimen) (in this format: _ _-_ _-_ _ _ _) |  |
|  | If other molecular tests were performed, what was the result (for each type of specimen)? |  |
| 56, 57, 58, 59, 60 | Has serology for SARS-CoV-2 antibodies been performed? | ( ) Yes ( ) No ( ) Unknown |
|  | If yes, specify the type of serology test (ELISA/IFA, NA, Other) |  |
|  | If yes, specify the type of specimen | ( ) Serum ( ) Other |
|  | If yes, which date was the specimen taken? | _ _-_ _-_ _ _ _ |
|  | If yes, what was the result? |  |
| 61 | Abnormal lung X-ray | ( ) Yes ( ) No ( ) Unknown |
| Respiratory diagnostic testing | | |
| 62, 63 | Other respiratory pathogens tested (than SARS-CoV-2)? | ( ) Yes ( ) No |
|  | If yes, which of the following pathogen(s) was detected? | ( ) Influenza (rapid Ag test)  ( ) Influenza A/B (PCR A/B test)  ( ) RSV (Respiratory Syncytial Virus)  ( ) Human Metapneumovirus  ( ) Parainfluenza virus (1-4)  ( ) Adenovirus  ( ) Rhinovirus/Enterovirus  ( ) Coronavirus (OC43, 229E, HKU1, NL63)  ( ) Mycobacterium Pneumoniae  ( ) Chlamydia pneumoniae  ( ) Other, specify: |
| Secondary bacterial infection | | |
| 64, 65, 66, 67 | Was a secondary infection detected? | ( ) Yes ( ) No ( ) Unknown |
|  | If yes, date of sample | _ _-_ _-_ _ _ _ |
|  | If yes, type of sample | ( ) Sputum  ( ) Endotracheal aspirate  ( ) Pleural fluid  ( ) CSF (cerebrospinal fluid)  ( ) Blood  ( ) Urine  ( ) Faeces  ( ) Other, specify: |
|  | If yes, which of the following pathogens was detected? | ( ) Haemophilus influenzae  ( ) Staphylococcus aureus  ( ) Streptococcus pneumoniae  ( ) Escherichia coli  ( ) Other organism, specify: |
