# Supplementary figures and images for: A prospective cohort study of SARS-CoV-2 infection-induced seroconversion and disease incidence in German healthcare workers before and during the rollout of COVID-19 vaccines
Source: PLoS One. 2024 Jan 30;19(1):e0294025. doi: 10.1371/journal.pone.0294025 (PMC10826949; doi:10.1371/journal.pone.0294025)

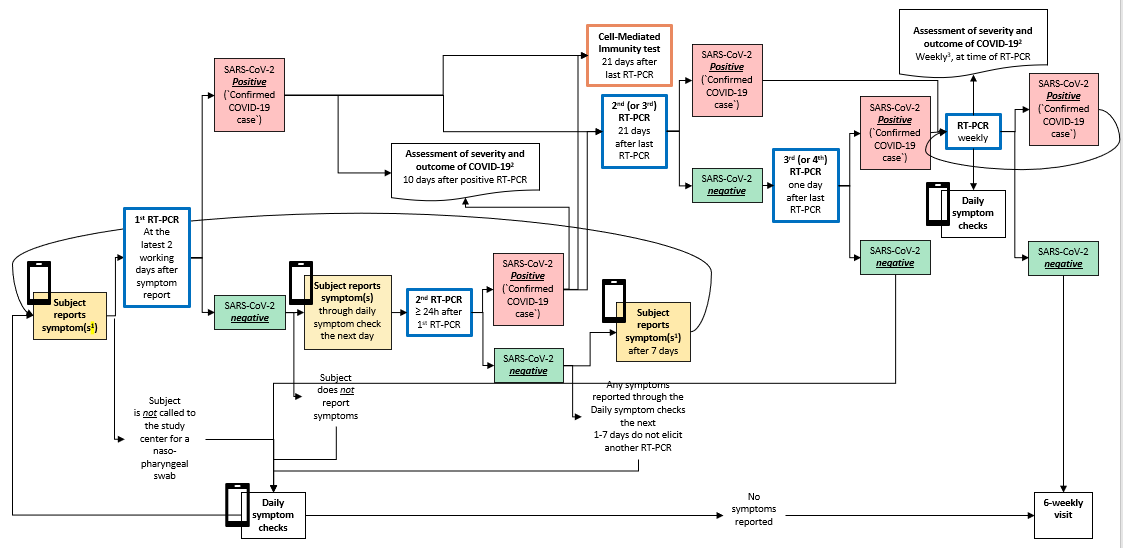

Supplement: S1 Fig — (DOCX) [file pone.0294025.s003.docx]

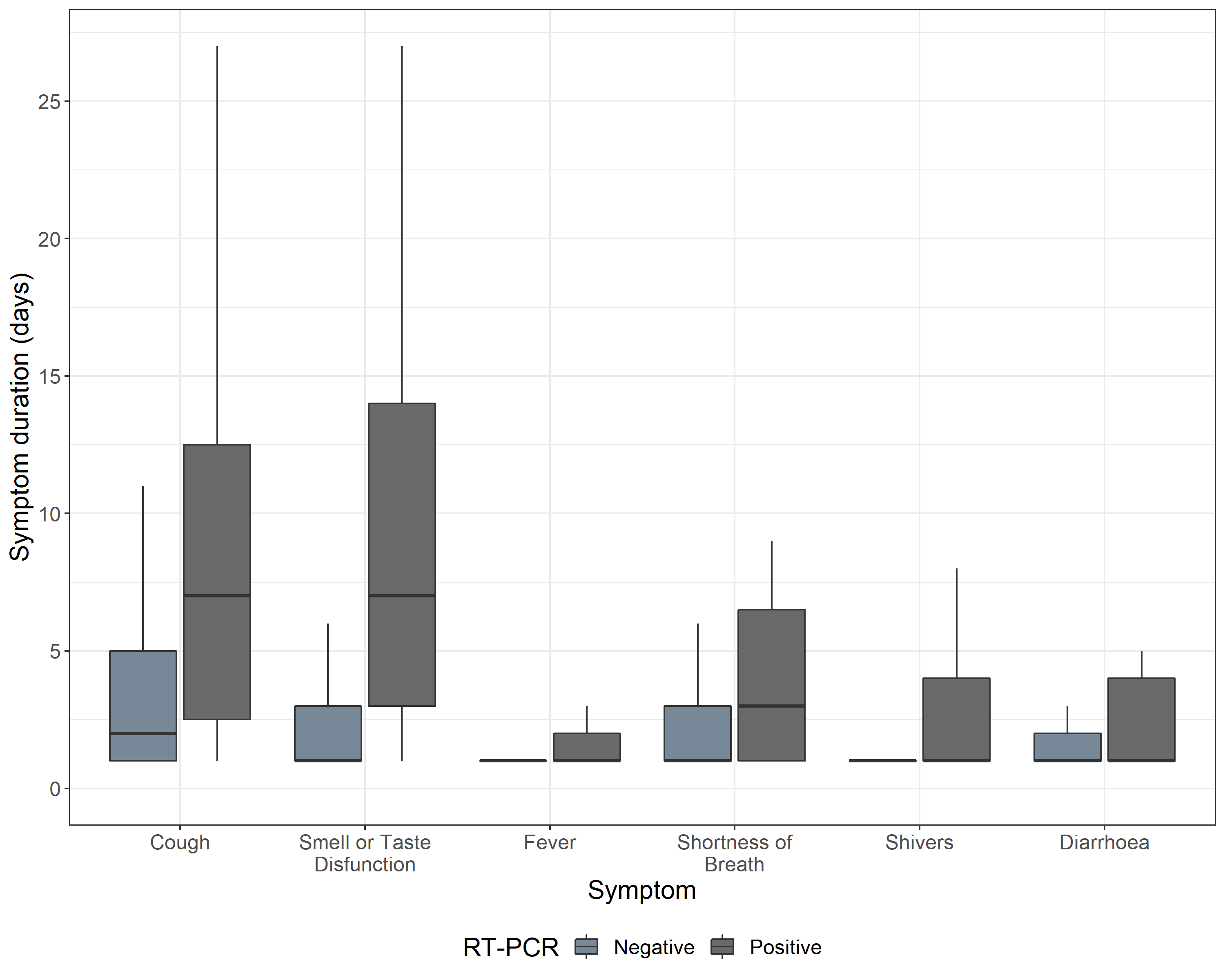


**S12 Figure. Duration of clinical symptoms of suspected COVID-19 cases**

Supplement: S4 Fig — (DOCX) [file pone.0294025.s006.docx]
