# Supplementary material for: A prospective cohort study of SARS-CoV-2 infection-induced seroconversion and disease incidence in German healthcare workers before and during the rollout of COVID-19 vaccines
Source: PLoS One. 2024 Jan 30;19(1):e0294025. doi: 10.1371/journal.pone.0294025 (PMC10826949; doi:10.1371/journal.pone.0294025)
Supplement: S2 Fig — (DOCX) [file pone.0294025.s004.docx]

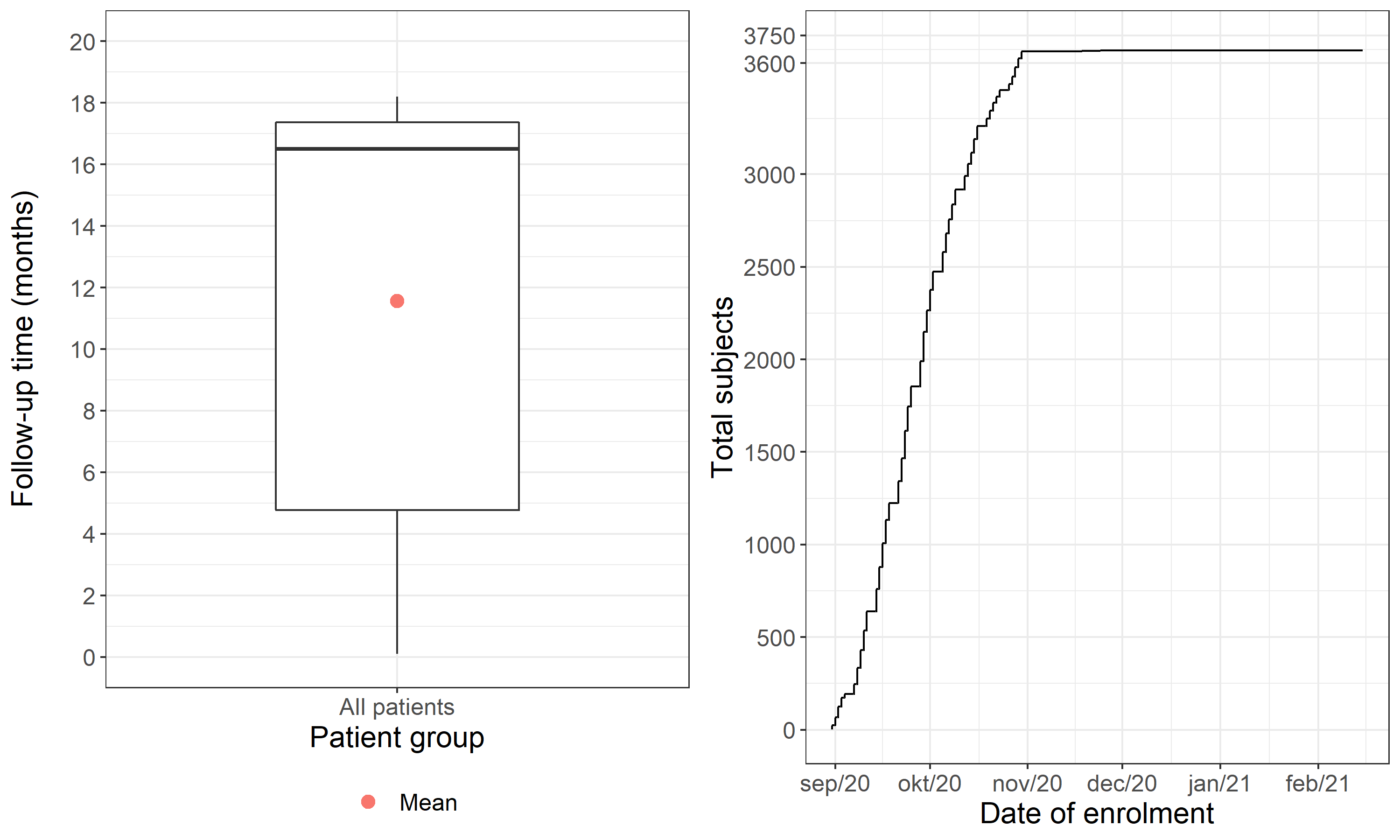


**S10 Figure A. Study enrolment and summary of follow-up duration.**


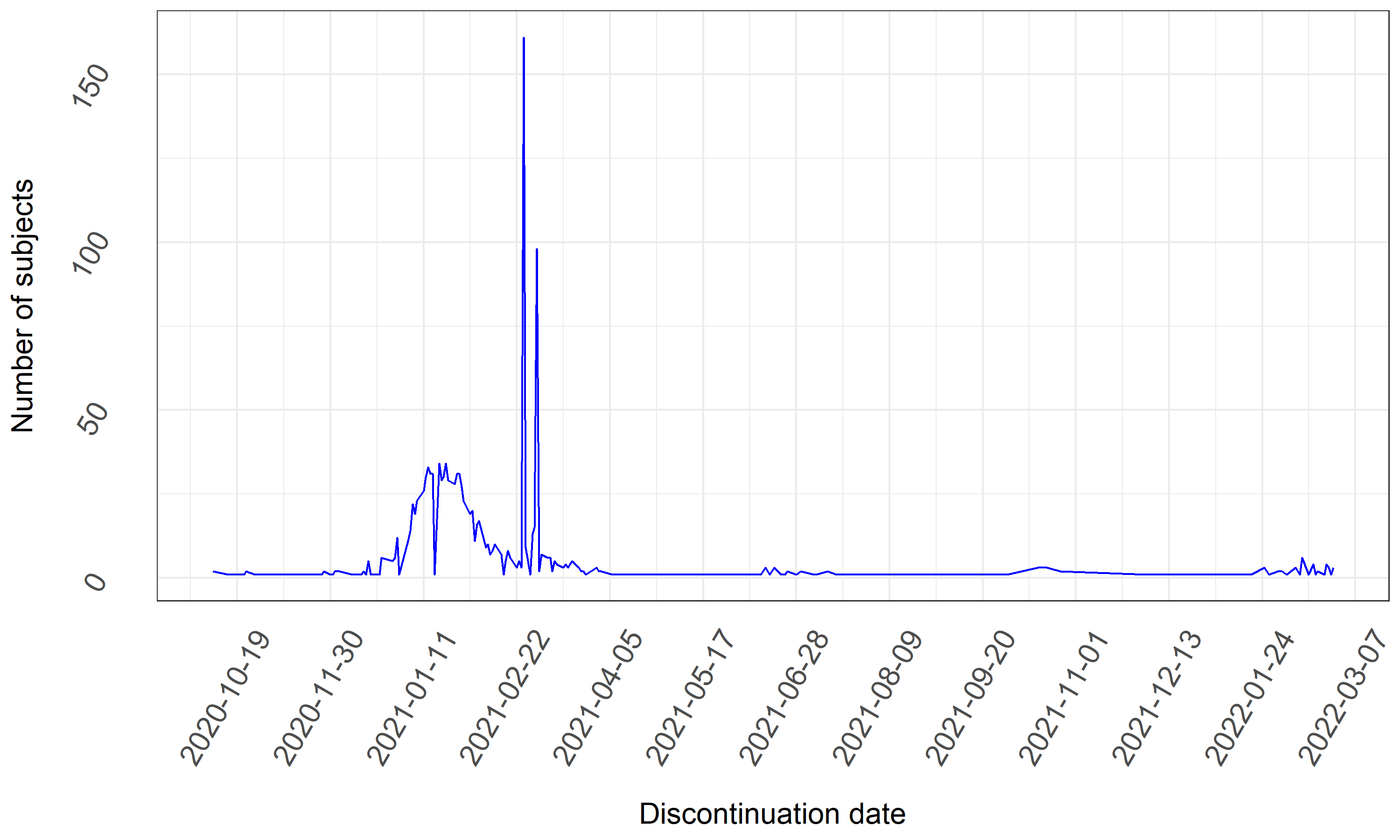


**S10 Figure B. Timing of discontinuation. The highest two successive peaks, at the beginning of January 2021 and in mid-February 2021, correspond to the time when most transfers to the vaccine trial occurred.**
