# Supplementary material for: A prospective cohort study of SARS-CoV-2 infection-induced seroconversion and disease incidence in German healthcare workers before and during the rollout of COVID-19 vaccines
Source: PLoS One. 2024 Jan 30;19(1):e0294025. doi: 10.1371/journal.pone.0294025 (PMC10826949; doi:10.1371/journal.pone.0294025)
Supplement: S3 Fig — (DOCX) [file pone.0294025.s005.docx]

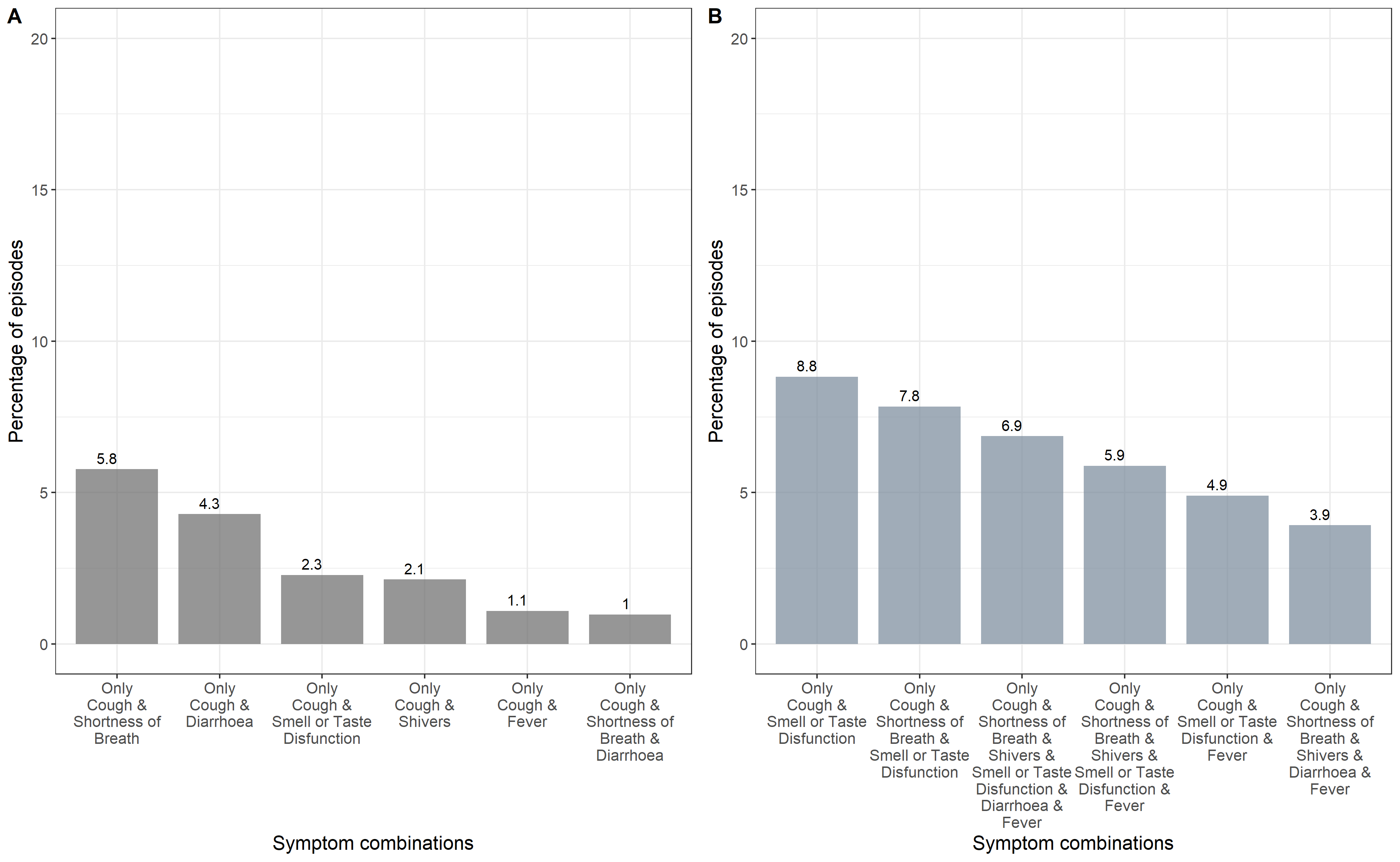


**S11 Figure. Combinations of clinical symptoms of suspected COVID-19 cases: A) Negative episodes, and B) Positive episodes.**
