# Supplementary material for: A prospective cohort study of SARS-CoV-2 infection-induced seroconversion and disease incidence in German healthcare workers before and during the rollout of COVID-19 vaccines
Source: PLoS One. 2024 Jan 30;19(1):e0294025. doi: 10.1371/journal.pone.0294025 (PMC10826949; doi:10.1371/journal.pone.0294025)
Supplement: S1 Table — (DOCX) [file pone.0294025.s007.docx]

| **FDA definition*** | **CEPI definition**** |
| --- | --- |
| Positive SARS-CoV-2 specific RT-PCR | Positive SARS-CoV-2 specific RT-PCR |
| **AND** | **AND** |
| acute illness with one or several of the following symptoms:   - fever ≥ 37.8°C or chills - cough - shortness of breath or difficulty breathing or fatigue - muscle or body aches - headache - new loss of taste or smell - sore throat - congestion or runny nose - nausea or vomiting - diarrhea | acute illness with symptoms from at least two of the following three categories: |
|  | 1. New onset LRTD as diagnosed by any one or more of the following signs and symptoms:   - persistent dry (non-productive) cough - dyspnea or tachypnea (RR > 20/min) - low peripheral capillary oxygen saturation (SpO2 < 95% on room air) as measured by pulse-oximetry - chest pain - radiographic findings consistent with LRTD |
|  | 2. Fever or history of new-onset fever (defined as a body temperature of ≥ 37.8°C irrespective of method) |
|  | 3. New onset systemic viral illness as diagnosed by any one or more of the following symptoms: a) myalgia, b) chills, c) loss of smell or taste, d) headache, e) sore throat, f) diarrhea |

# Virologically confirmed COVID-19 illness

FDA U.S. Food & Drug Administration

CEPI Coalition for Epidemic Preparedness Innovations

LRTD Lower respiratory tract disease

RR Respiratory rate

# Severe confirmed virologically confirmed COVID-19 illness

| **FDA definition** | **CEPI definition** |
| --- | --- |
| Positive SARS-CoV-2 specific RT-PCR | Positive SARS-CoV-2 specific RT-PCR |
| **AND** | **AND** |
| acute illness with one or several of the following symptoms:   - clinical signs at rest indicative of severe systemic illness (respiratory rate ≥ 30 per minute, heart rate ≥ 125 per minute, SpO2 ≤ 93% on room air at sea level, or PaO2/FiO2 < 300 mm Hg) - respiratory failure (defined as needing high-flow oxygen, noninvasive ventilation, mechanical ventilation, or ECMO) - evidence of shock (SBP < 90 mm Hg, DBP < 60 mm Hg, or requiring vasopressors) - significant acute renal, hepatic, or neurologic dysfunction - admission to an ICU - death | a NEWS2 score of >6, as follows*:  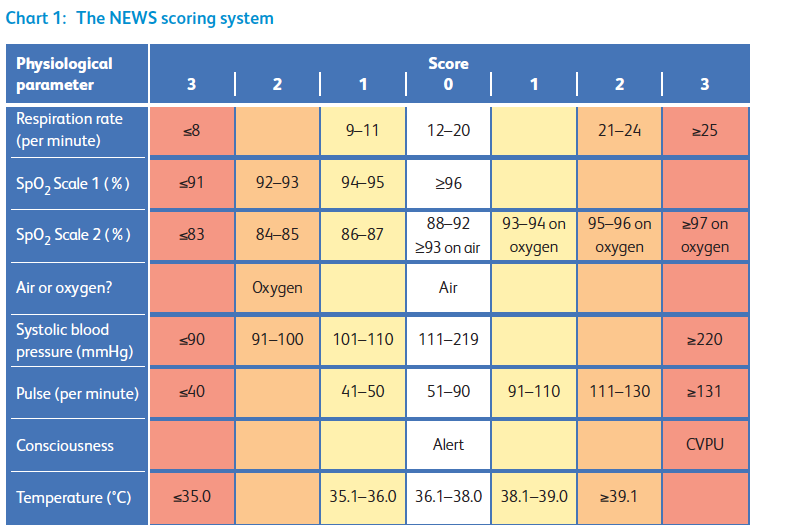 |

* <https://www.rcplondon.ac.uk/file/9434/download> (accessed: 25 July, 2022)

# Suspected COVID-19 disease

| Acute illness that is clinically consistent with COVID-19 based on the presence of any of the following symptoms:   - fever ≥ 37.8°C, - cough, - shortness of breath, - chills, and/or - loss of taste and/or smell. |
| --- |
