# Supplementary material for: A prospective cohort study of SARS-CoV-2 infection-induced seroconversion and disease incidence in German healthcare workers before and during the rollout of COVID-19 vaccines
Source: PLoS One. 2024 Jan 30;19(1):e0294025. doi: 10.1371/journal.pone.0294025 (PMC10826949; doi:10.1371/journal.pone.0294025)
Supplement: S2 Table — (DOCX) [file pone.0294025.s008.docx]

| Visit number | Number of subjects who completed the visit | Date of first subject’s visit | Date of last subject’s visit | Median date |
| --- | --- | --- | --- | --- |
| 1 | 3663 | 31-08-2020 | 18-11-2021 | 25-09-2020 |
| 2 | 3615 | 19-10-2020 | 29-01-2021 | 03-11-2020 |
| 3 | 3541 | 23-11-2020 | 29-06-2021 | 10-12-2020 |
| 4 | 3275 | 04-01-2021 | 23-02-2022 | 22-01-2021 |
| 5 | 2104 | 07-06-2021 | 25-02-2022 | 18-06-2021 |
| 6 | 1880 | 12-01-2022 | 25-02-2022 | 09-02-2022 |
