# Supplementary material for: A prospective cohort study of SARS-CoV-2 infection-induced seroconversion and disease incidence in German healthcare workers before and during the rollout of COVID-19 vaccines
Source: PLoS One. 2024 Jan 30;19(1):e0294025. doi: 10.1371/journal.pone.0294025 (PMC10826949; doi:10.1371/journal.pone.0294025)
Supplement: S3 Table — (DOCX) [file pone.0294025.s009.docx]

| Observation number (scheduled visit) | 1* | 2 | 3 | 4 | 5 | 6 |
| --- | --- | --- | --- | --- | --- | --- |
| Signed informed consent (all subjects, at study site) | X |  |  |  |  |  |
| Subject information and pseudonymization  (all subjects, at study site) | X | X | X | X | X | X |
| Inclusion/exclusion criteria (all subjects) | X |  |  |  |  |  |
| Questionnaire on demographics/risk factors  (all subjects, at study site) | X |  |  |  |  |  |
| Symptom check (all subjects, app/paper) | On a daily/biweekly basis throughout the study period | | | | | |
| Scheduled visit questionnaire on risk factors  (all subjects, app/paper) | X | X | X | X | X | X |
| Confirmed case questionnaire  (for RT-PCR positive cases, at study site by study staff) | Only for suspect COVID-19 cases who are RT-PCR positive | | | | | |
| Serology: antibodies to SARS-CoV-2 | X | X | X | X | X | X |
| Nasopharyngeal swab: RT-PCR for SARS-CoV-2 | Following the algorithm throughout the  study period | | | | | |

* First contact
