# Supplementary material for: A prospective cohort study of SARS-CoV-2 infection-induced seroconversion and disease incidence in German healthcare workers before and during the rollout of COVID-19 vaccines
Source: PLoS One. 2024 Jan 30;19(1):e0294025. doi: 10.1371/journal.pone.0294025 (PMC10826949; doi:10.1371/journal.pone.0294025)
Supplement: S4 Table — (DOCX) [file pone.0294025.s010.docx]

|  | Visit 1 (enrollment visit) | Visit 2 | Visit 3 | Visit 4 | Visit 5 | Visit 6 (last visit) |
| --- | --- | --- | --- | --- | --- | --- |
| IgG  (anti-N)* | X | X | X | X | X | X |
| IgG  (anti-S)** |  |  |  |  | X | X |
| IgM  (anti-S)*** | X | X | X | X |  |  |

* Tested using the assay ARCHITECT® i2000SR system from Abbott Laboratories (test sensitivity 100% and specificity 99.6%). This test is considered specific to infection-induced immunity (shaded cells). Since COVID-19 vaccines used in Germany are only antigenic to S protein (i.e., only elicit anti-S antibodies), study subjects who carry anti-N antibodies are assumed to have had a natural infection. If positive or borderline-positive, samples were re-tested using the Elecsys® Anti-Anti-N SARS-CoV-2 IgG/IgM by Roche Diagnostics (test sensitivity 99.5% and specificity 99.8%).

** Tested using the assay ᾿SARS-CoV-2 IgG II Quant᾿ from Abbott Laboratories (test sensitivity 98.3% and specificity 99.4%). A positive result may indicate vaccination and/or infection-induced immunity (shaded cells indicate when COVID-19 vaccination may have occurred).

*** Tested using the assay ᾿AdviseDx SARS-CoV-2 IgM᾿ from Abbott Laboratories (test sensitivity 95% and specificity 99.6%). A positive result may indicate vaccination and/or infection-induced immunity (shaded cells indicate when COVID-19 vaccination may have occurred).
