# Supplementary material for: A prospective cohort study of SARS-CoV-2 infection-induced seroconversion and disease incidence in German healthcare workers before and during the rollout of COVID-19 vaccines
Source: PLoS One. 2024 Jan 30;19(1):e0294025. doi: 10.1371/journal.pone.0294025 (PMC10826949; doi:10.1371/journal.pone.0294025)
Supplement: S5 Table — (DOCX) [file pone.0294025.s011.docx]

The table below shows the characteristics of the study population, stratified by seroconversion status.

| Characteristics | Age category (years) | Subjects that seroconverted for anti-N IgG (visits 2-6) and/or anti-S IgM (visits 2-3) (n=255):  Number (%) | Subjects that never seroconverted  (n=1661):  Number (%) | p-value |
| --- | --- | --- | --- | --- |
| Age group (years) | 17 - 29 | 121 (47.5%) | 473 (28.5%) | 0.06 |
|  | 30 - 39 | 53 (20.8%) | 435 (26.2%) |  |
|  | 40 - 49 | 38 (14.9%) | 269 (16.2%) |  |
|  | 50 - 59 | 32 (12.5%) | 327 (19.7%) |  |
|  | 60 – 77.5 | 11 (4.3%) | 157 (9.4%) |  |
| Sex | Female | 192 (75.3%) | 1192 (71.7%) | 0.69 |
|  | Male | 63 (24.7%) | 468 (28.2%) |  |
|  | Other | 0 (0.0%) | 1 (0.1%) |  |
| Occupational group | Doctor | 28 (11.0%) | 218 (13.1%) | 0.15 |
|  | Nursing | 86 (33.7%) | 413 (24.9%) |  |
|  | Student | 60 (23.5%) | 270 (16.2%) |  |
|  | Other | 81 (31.8%) | 760 (45.7%) |  |
| Smoker | No | 219 (85.9%) | 1405 (84.6%) | 0.98 |
|  | Yes | 36 (14.1%) | 253 (15.2%) |  |
| Disorders of airways and/or lung | No | 234 (91.8%) | 1517 (91.3%) | 1.00 |
|  | Yes | 21 (8.2%) | 139 (8.4%) |  |
| Disorders of cardiovascular system | No | 240 (94.1%) | 1530 (92.1%) | 0.79 |
|  | Yes | 15 (5.9%) | 130 (7.8%) |  |
| Immune deficiency | No | 251 (98.4%) | 1629 (98.1%) | 1.00 |
|  | Yes | 4 (1.6%) | 29 (1.7%) |  |
| Direct care for SARS-CoV-2 patients | No | 216 (84.7%) | 1489 (89.6%) | 0.40 |
|  | Yes | 39 (15.3%) | 171 (10.3%) |  |
| Aware of contact with a possible SARS-CoV-2 patient | No | 226 (88.6%) | 1524 (91.7%) | 0.61 |
|  | Yes | 29 (11.4%) | 137 (8.3%) |  |
| Possibly been in a region with known SARS-CoV-2 transmission | No | 4 (1.6%) | 29 (1.7%) | 1.00 |
|  | Yes | 251 (98.4%) | 1629 (98.1%) |  |
| Swab/Follow-up test for SARS-CoV-2 prior to the study | No | 173 (67.8%) | 1152 (69.3%) | 0.93 |
|  | Yes | 82 (32.2%) | 507 (30.6%) |  |
