# Supplementary material for: A prospective cohort study of SARS-CoV-2 infection-induced seroconversion and disease incidence in German healthcare workers before and during the rollout of COVID-19 vaccines
Source: PLoS One. 2024 Jan 30;19(1):e0294025. doi: 10.1371/journal.pone.0294025 (PMC10826949; doi:10.1371/journal.pone.0294025)
Supplement: S6 Table — (DOCX) [file pone.0294025.s012.docx]

| Reasons for study discontinuation | Number of subjects | % |
| --- | --- | --- |
| Switching to COVID-19 vaccine trial | 817 | 66.59% |
| Lost to follow-up | 263 | 21.43% |
| Miscellaneous reasons | 87 | 7.09% |
| Withdrawn | 57 | 4.65% |
| Serious protocol deviation | 2 | 0.16% |
| Significant clinical deterioration | 1 | 0.08% |
| Total number of discontinuations | 1227 | 100.00% |
