# Supplementary material for: A prospective cohort study of SARS-CoV-2 infection-induced seroconversion and disease incidence in German healthcare workers before and during the rollout of COVID-19 vaccines
Source: PLoS One. 2024 Jan 30;19(1):e0294025. doi: 10.1371/journal.pone.0294025 (PMC10826949; doi:10.1371/journal.pone.0294025)
Supplement: S7 Table — (DOCX) [file pone.0294025.s013.docx]

**Baseline characteristics, including demographics and COVID-19 disease risk factors, among healthcare workers at Universitätsmedizin Mainz, Germany, stratified by vaccination status**

| **Characteristics** | **Category** | **All participants at enrollment**  **(N=3665)** **n (%)** | **Participants with full vaccination**  **(N=1966)**  **n (%)** | **Participants with partial vaccination**  **(N=39)**  **n (%)** | **Unvaccinated participants**  **(N = 50)**  **N (%)** | **p-value** |
| --- | --- | --- | --- | --- | --- | --- |
| Age group (years) | 17-29 | 1110 (30.3) | 611 (31.1) | 8 (20.5) | 11 (22.0) | 0.16 |
|  | 30 - 39 | 898 (24.5) | 449 (22.8) | 13 (33.3*)* | 16 (32.0) |  |
|  | 40 - 49 | 601 (16.4) | 335 (17.0) | 7 (17.9) | 7 (14.0) |  |
|  | 50 - 59 | 720 (19.6) | 392 (19.9) | 5 (12.8) | 12 (24.0) |  |
|  | 60-80 | 336 (9.2) | 179 (9.1) | 6 (15.4) | 4 (8.0) |  |
| Sex | Female | 2761 (75.3) | 1559 (79.3) | 27 (69.2) | 37 (74.0) | 0.58 |
|  | Male | 901 (24.6) | 405 (20.6) | 12 (30.8) | 13 (26.0) |  |
|  | Other | 3 (0.1) | 2 (0.1) | 0 (0.0) | 0 (0.0) |  |
| Occupational group | Doctor | 529 (14.4) | 295 (15) | 2 (5.1) | 8 (16.0) | 0.08 |
|  | Nurse | 1083 (29.5) | 659 (33.5) | 16 (41) | 23 (46.0) |  |
|  | Student | 608 (16.6) | 323 (16.4) | 6 (15.4) | 5 (10.0) |  |
|  | Other | 1445 (39.4) | 689 (35) | 15 (38.5) | 14 (28.0) |  |
| Smoker* | No | 3110 (84.9) | 1676 (85.2) | 31 (79.5) | 41 (82.0) | 0.56 |
|  | Yes | 552 (15.1) | 290 (14.8) | 8 (20.5) | 9 (18.0) |  |
| Disorders of airways and/or lung ** | No | 3315 (90.5) | 1763 (89.7) | 34 (87.2) | 45 (90.0) | 0.78 |
|  | Yes | 344 (9.4) | 202 (10.3) | 5 (12.8) | 5 (10.0) |  |
| Disorders of cardiovascular system*** | No | 3362 (91.7) | 1793 (91.2) | 35 (89.7) | 46 (92.0) | 0.85 |
|  | Yes | 301 (8.2) | 172 (8.7) | 4 (10.3) | 4 (8.0) |  |
| Immune deficiency**** | No | 3568 (97.4) | 1900 (96.6) | 37 (94.9) | 49 (98.0) | 0.48 |
|  | Yes | 94 (2.6) | 66 (3.4) | 2 (5.1) | 1 (2.0) |  |
| Direct care for SARS-CoV-2 patients | No | 3132 (85.5) | 1615 (82.1) | 37 (94.9) | 41 (82.0) | 0.01 |
|  | Yes | 528 (14.4) | 347 (17.7) | 2 (5.1) | 9 (18.0) |  |
| Aware of contact with a possible SARS-CoV-2 patient | No | 3296 (89.9) | 1745 (88.8) | 38 (97.4) | 43 (86.0) | 0.01 |
|  | Yes | 369 (10.1) | 221 (11.2) | 1 (2.6) | 7 (14.0) |  |
| Possibly been in a region with known SARS-CoV-2 transmission++ | No | 94 (2.6) | 66 (3.4) | 2 (5.1) | 1 (2.0) | 0.48 |
|  | Yes | 3568 (97.4) | 1900 (96.6) | 37 (94.9) | 49 (98.0) |  |
| Swab/follow-up test for SARS-CoV-2 prior to the study+++ | No | 2501 (68.2) | 1324 (67.3) | 21 (53.8) | 31 (62.0) | 0.14 |
|  | Yes | 1161 (31.7) | 641 (32.6) | 18 (46.2) | 19 (38.0) |  |

*: 3, 1, and 2 participant(s) with missing information regarding smoking status, respectively for all participants, regular end participants, and discontinued participants.

**: 6, 4, and 2 participants with missing information regarding disorders of airways and/or lungs, respectively for all participants, regular end participants, and discontinued participants.

***: 2, 1, and 1 participant(s) with missing information regarding disorders of the cardiovascular system, respectively for all participants, regular end participants, and discontinued participants.

****: 3, 2, and 1 participant(s) with missing information regarding immune deficiency, respectively for all participants, regular end participants, and discontinued participants.

+: 5, 4, and 1 participant(s) with missing information regarding direct care for COVID-19 patients, respectively for all participants, regular end participants, and discontinued participants.

++: 3, 2, and 1 participant(s) with missing information regarding possibly having been in a region with known SARS-CoV-2 transmission, respectively for all participants, regular end participants, and discontinued participants.

+++: 3, 2, and 1 participant(s) with missing information regarding swab/follow-up test for SARS-CoV-2 prior to the study, respectively for all participants, regular end participants, and discontinued participants.
