# Supplementary material for: A prospective cohort study of SARS-CoV-2 infection-induced seroconversion and disease incidence in German healthcare workers before and during the rollout of COVID-19 vaccines
Source: PLoS One. 2024 Jan 30;19(1):e0294025. doi: 10.1371/journal.pone.0294025 (PMC10826949; doi:10.1371/journal.pone.0294025)
Supplement: S8 Table — (DOCX) [file pone.0294025.s014.docx]

**Crude and adjusted seroprevalence of anti-SARS-CoV-2 antibodies at scheduled visits 3-5, stratified by age/sex/comorbidities and vaccine uptake**

We performed an exploratory analysis to see if the seroprevalence of anti-SARS-CoV-2 antibodies at the scheduled visits was different with respect to age, sex, comorbidities, and vaccine uptake of participants. There were five age groups: ≤29, 30-39, 40-49, 50-59, and ≥60 years. We only considered participants with sex either Male (`M`) or Female (`F`), i.e., we discarded three observations whose sex was defined as `Other`.

Participants were categorized as having comorbidities if they had indicated “Yes” (comorbidity == 1) to any of the questions related to *disorders of airways and/or lung, disorders of the cardiovascular system* or *immune deficiency.* Participants were categorized as `Vaccinated` (vaccine_update == 1) if they received at least one vaccine dose prior to the date of visit. If they did not, they were categorized as `Unvaccinated` (vaccine_uptake == 0).

The exploratory analysis showed that the seroprevalence of anti-SARS-CoV-2 antibodies at scheduled visit 3 should be reported by age group. Seroprevalence at visit 4 should be reported by age group and vaccine update. Seroprevalence at visit 5 should be stratified by sex and vaccine uptake. There was no need to stratify the data by visits 1-2 and 6. We thus show the seroprevalence results by different strata at visits 3-5 in Panels A, B, and C, respectively.

**Panel A: Crude and adjusted seroprevalence of anti-SARS-CoV-2 antibodies at scheduled visit 3, stratified by age groups**

| **Visit 3** | **Age group (years)** | | | | |
| --- | --- | --- | --- | --- | --- |
|  | **≤ 29** | **30-39** | **40-49** | **50-59** | **≥60** |
| Total number of participants with serostatus data | 1068 | 852 | 586 | 707 | 325 |
| Total number of participants with positive serostatus | 69 | 28 | 23 | 24 | 8 |
| Crude seroprevalence  % [95% CI] | 6.5  [5.1; 8.1] | 3.3  [2.2; 4.7] | 3.9  [2.5; 5.8] | 3.4  [2.2; 5.0] | 2.5  [1.1; 4.8] |
| Adjusted seroprevalence  % [95% CI] | 5.8  [5.3; 6.3] | 2.6  [2.2; 3.0] | 3.3  [2.7; 3.8] | 2.7  [2.3; 3.2] | 2.0  [1.3; 2.5] |

**Panel B: Crude and adjusted seroprevalence of anti-SARS-CoV-2 antibodies at scheduled visit 4, stratified by age groups and vaccine uptake**

| **Visit 4** | **Age group (years)** | | | | |
| --- | --- | --- | --- | --- | --- |
|  | **≤ 29** | **30-39** | **40-49** | **50-59** | **≥60** |
| **Unvaccinated participants** | | | | | |
| Total number of participants with serostatus data | 842 | 642 | 461 | 558 | 250 |
| Total number of participants with positive serostatus | 82 | 44 | 30 | 30 | 11 |
| Crude seroprevalence  % [95% CI] | 9.4  [7.8; 11.9] | 6.9  [5.0; 9.1] | 6.5  [4.4; 9.2] | 5.4  [3.7; 7.6] | 4.4  [2.2; 7.7] |
| Adjusted seroprevalence  % [95% CI] | 9.1  [8.4; 9.8] | 6.2  [5.5; 6.9] | 5.9  [5.1; 6.7] | 4.8  [4.1; 5.4] | 4.0  [3.0; 4.8] |
| **Vaccinated participants** | | | | | |
| Total number of participants with serostatus data | 137 | 138 | 93 | 105 | 47 |
| Total number of participants with positive serostatus | 65 | 46 | 30 | 23 | 13 |
| Crude seroprevalence  % [95% CI] | 47.4  [38.9; 56.1] | 33.3  [25.5; 41.9] | 32.3  [22.9; 42.7] | 21.9  [14.4; 31.0] | 27.7  [15.6; 42.6] |
| Adjusted** seroprevalence  % [95% CI] | 47.1  [44.2; 49.9] | 33.0  [30.3; 35.7] | 32.1  [28.8; 35.3] | 21.8  [19.0; 24.5] | 28.0  [23.4; 32.2] |

**Panel C: Crude and adjusted seroprevalence of anti-SARS-CoV-2 antibodies at scheduled visit 5, stratified by sex and vaccine uptake**

| **Visit 5** | **Males** | | **Females** | |
| --- | --- | --- | --- | --- |
|  | **Unvaccinated** | **Vaccinated** | **Unvaccinated** | **Vaccinated** |
| Total number of participants with serostatus data | 72 | 301 | 203 | 1221 |
| Total number of participants with positive*serostatus | 56 | 293 | 106 | 1177 |
| Crude seroprevalence  % [95% CI] | 77.8  [66.4; 86.7] | 97.3  [94.8; 98.8] | 52.2  [45.1; 59.3] | 96.4  [95.2; 97.4] |
| Adjusted seroprevalence  % [95% CI] | 76.8  [73.7; 80.3] | 97.0  [96.4; 97.7] | 51.8  [49.5; 54.2] | 96.3  [95.9; 96.7] |
